# Supplementary material for: Developing a dynamic model of anomalous experiences and function in young people with or without psychosis: a cross-sectional and longitudinal study protocol
Source: BMJ Open. 2018 Nov 3;8(11):e022546. doi: 10.1136/bmjopen-2018-022546 (PMC6231554; doi:10.1136/bmjopen-2018-022546)
Supplement: Author's manuscript [file bmjopen-2018-022546.draft_revisions.pdf]

BMJ Open is committed to open peer review. As part of this commitment we make the peer review history of every article we publish publicly available.

When an article is published we post the peer reviewers' comments and the authors' responses online. We also post the versions of the paper that were used during peer review. These are the versions that the peer review comments apply to.

The versions of the paper that follow are the versions that were submitted during the peer review process. They are not the versions of record or the final published versions. They should not be cited or distributed as the published version of this manuscript.

BMJ Open is an open access journal and the full, final, typeset and author-corrected version of record of the manuscript is available on our site with no access controls, subscription charges or pay-per-view fees (http://bmjopen.bmj.com).

If you have any questions on BMJ Open's open peer review process please email info.bmjopen@bmj.com

BMJ Open

Developing a dynamic model of unusual experiences and function in young people with or without psychosis: a cross-sectional and longitudinal study protocol.

Journal:	*BMJ Open*
Manuscript ID	bmjopen-2018-022546
Article Type:	Protocol
Date Submitted by the Author:	23-Feb-2018

Complete List of Authors:	Wright, Abigail; University of Sussex, School of Psychology<br>Fowler, David; University of Sussex, School of Psychology<br>Greenwood, Kathryn; University of Sussex, School of Psychology; Sussex Partnership NHS Foundation Trust

Keywords:	Schizophrenia & psychotic disorders < PSYCHIATRY, PSYCHIATRY, Anomalous experiences, Functional outcome, Subjective outcome, Cognition

SCHOLARONE™
Manuscripts

For peer review only

Developing a dynamic model of unusual experiences and function in young people with or without psychosis: a cross-sectional and longitudinal study protocol.

Abigail Wright* [a], Professor David Fowler [a b], Dr Kathryn Greenwood [a b]*.

[a] *University of Sussex, School of Psychology*

[b] *Sussex Partnership NHS Foundation Trust*

correspondence to Abigail Wright: a.c.wright@sussex.ac.uk

For peer review only

ABSTRACT

Introduction: Anomalous experiences are common within the general population, but the frequency and intensity is increased in young people with psychosis. Studies have demonstrated that perceptual biases towards noticing these phenomena plays a role, but the way one thinks about one's experience (metacognition) may also be relevant. Whilst poor metacognitive function has been theoretically associated with anomalous experiences, this relationship is currently unclear. However, metacognition may work along a continuum with various metacognitive levels, many of which have been demonstrated as impaired in psychosis. These metacognitive components may interact via processes that maintain poor metacognition across levels, and that potentially impact both what people do in their everyday lives (functional outcome) and how people feel about their everyday lives (subjective recovery outcome) in young people with psychosis compared to healthy control participants.

Methods and analysis: This cross-sectional study will investigate the association and contribution of metacognition to anomalous experiences and outcome measures cross-sectionally, and longitudinally in a 36-month follow-up. Firstly, young people with psychosis will be compared with healthy control participants on selected measures of anomalous experience, metacognition, and function, using ANCOVA to identify group differences. Next, the relationship between metacognitive components and processes will be explored, including processes connecting the different components, using regression analyses. Finally, mediation analyses will be used to assess the predictive value of metacognitive measures on outcome measures, cross-sectionally and at 36-months, whilst controlling for symptoms and cognition.

For peer review only - http://bmjopen.bmj.com/site/about/guidelines.xhtml

Ethics and dissemination: Ethical and Health Research Authority approval has been obtained through Camberwell St. Giles Research Ethics Committee (reference number: 17/LO/0055). This research project will be reported within a PhD thesis and submitted for journal publication. Once key predictive components of poor outcome in psychosis are identified, this study will develop a series of dynamic models to understand influences on outcome for young people with psychosis.

ARTICLE SUMMARY:

Strengths and limitations of this study:

- This is one of the first studies to assess metacognitive processes as predictors of functional outcome in young people with psychosis, and one of the first to assess metacognition as a longitudinal predictor of functional outcome in young people with psychosis sample.
- This study has used up-to-date paradigm within the field of metacognition to avoid biases.
- Due to length of follow-up period, it may be difficult to engage follow-up participants within this study. If re-contacting is difficult, the study may only have power to detect effects of the separate paths of the longitudinal analyses, not the full model.

Word count: 4839

INTRODUCTION

Anomalous experiences, ranging from the sense that you are not "real" (anomalous self-experiences; distortions in experience of self and being) to hearing sounds which cannot be accounted for by the environment (anomalous perceptual experiences: distortions of sensory events in various domains; auditory, visual; touch; taste) are commonly experienced by individuals within the general population (Bell, Halligan, & Ellis, 2006; Kelleher et al., 2012). However, the frequency and intensity of these unusual experiences is increased in those with psychosis, or those with emerging severe mental health difficulties (Brett, Johns, Peters, & McGuire, 2009; Reininghaus et al., 2016). Such experiences are suggested to be a precursor to full psychotic symptoms, e.g. hearing voices, and increasingly distressing experiences.

For peer review only - http://bmjopen.bmj.com/site/about/guidelines.xhtml

Many studies have demonstrated that anomalous perceptual experiences are associated with perceptual biases (Bentall & Slade, 1985; Kok, Kouider, Lange, & Supe, 2015). Perceptual biases are noted when an individual has a bias towards stating that a stimulus was present when it was in-fact absent. Such top-down biases have been consistently demonstrated within the psychosis literature (Mussgay & Hertwig, 1990; Teufel et al., 2015; Teufel, Kingdon, Ingram, Wolpert, & Fletcher, 2010), regardless of perceptual sensitivity: ability to detect stimulus (Mintz & Alpert, 1972; Bentall & Slade, 1985).

Recent evidence suggests these top-down biases are associated with metacognition in healthy students (Sherman, Seth, Barrett, & Kanai, 2015), and metacognition may therefore play a role in anomalous experiences. Metacognition is defined as “thinking about thinking” (Semerari et al., 2003) and the way one thinks about one’s experience. Nelson and Narens (1990) outlined a metacognitive model suggesting an object-level which includes cognitive processes (such as perception and memory) and a meta-level which is an abstract view of the object-level (deemed metacognition). It may be suggested that top-down biases predispose an individual to anomalous experiences. Then poor ability to accurately interpret and understand experiences and knowledge (metacognition) may play a role in the presence and maintenance of anomalous events. In such that if you are unable to accurately discriminate between a real or false perception, you may accept a false one as real. After this, if you are then overconfident in your errors, this may result in the maintenance of this false perception or development of anomalous delusional belief associated with the perception, e.g. delusional ideation. Literature has demonstrated that hallucinatory experiences and delusional beliefs/ideation have been associated with metacognition (overconfidence, specifically) in both clinical and non-clinical groups (Eisenacher et al., 2015; Moritz et al., 2005; Warman, 2008).

However, literature highlights that metacognition may be fractionated and appear in many different forms, associated within a dynamic model (Nelson & Narens, 1990; Shea et al., 2014). Three levels of metacognition have been proposed: i) Metacognitive efficiency: “knowing that you know” (Sherman, Barrett, & Kanai, 2015), and this level in particular may involve unconscious knowledge to generate a “feeling of knowing” (Nelson, 1984) and has been shown to be modality-specific (Fleming et al., 2014), which can be assessed by within-task confidence ratings, ii) Metacognitive experience: appraisal of one’s experience or performance after an activity, and iii) Metacognitive knowledge: trait-like, stable beliefs about one’s experience or abilities, on which Lysaker and colleagues have grounded their

For peer review only - http://bmjopen.bmj.com/site/about/guidelines.xhtml

work (see Lysaker et al. 2014). These metacognitive levels may influence each other via metacognitive processes. Nelson and Narens (1990) highlighted two processes: i) Metacognitive controlling processes, (i.e. such that knowledge is used to control, guide and correct ongoing action, for example, by shifting attentional focus Wells & Purdon, 1999), and ii) Metacognitive monitoring processes (i.e. monitoring of ongoing experience in order to recognise anomalies and update higher level beliefs (Efklides, 2006), which are important for ensuring accurate metacognitive functioning.

Research has demonstrated an association between anomalous experiences or delusional beliefs and metacognitive efficiency (within-task confidence) ratings (Moritz et al., 2005), although this research has not always been consistent (see Gawęda et al., 2013). This may be because studies have generally not controlled for objective performance whilst assessing metacognitive efficiency (Balzan et al., 2016), which is crucial within the cognitive literature (Fleming & Lau, 2014). In addition, associations may be more modality-specific than once thought as both metacognitive efficiency (Fleming et al., 2014; Morales, Lau & Fleming, 2017) and anomalous experiences (Mitchell et al., 2017) are modality specific. This present study will assess the modality-specific associations in the visual and auditory domains of metacognition (and confirm literature demonstrating association with perceptual bias outlined above) and anomalous experience, whilst controlling for objective performance.

Anomalous delusional beliefs are unusual, mistaken beliefs, ranging from paranoia (feeling as if others may have it in for you) to persecutory delusions (wrongly believing that others are out to harm or conspire against you) or grandiose delusions (feeling as if you are God). Anomalous delusional beliefs, such as paranoia, are commonly experienced by those with psychosis, those considered at high-risk, and those within the general population (Hodgekins et al., 2012; Lower et al., 2015). Research has suggested that anomalous delusional beliefs may be developed from anomalous experiences (Fletcher & Frith, 2009; Garety et al., 2001; Freeman et al., 2002) signifying a hierarchical framework for anomalous experiences to anomalous delusional beliefs. Several studies have also recently demonstrated that delusional beliefs (including paranoia) were significantly associated with metacognitive efficiency, within a sample of individuals with schizophrenia (Moritz et al., 2014), although, again, the associations were not consistent (see Kircher et al., 2007). This study will assess the relationship between anomalous delusional beliefs and metacognitive efficiency.

For peer review only

For peer review only - http://bmjopen.bmj.com/site/about/guidelines.xhtml

Finally, anomalous self-experiences are also suggested to be associated with anomalous experiences as they may be considered the underlying disturbance preceding and generating "surface-level" anomalous experiences (Nelson, Parnas & Sass, 2014). This suggests a hierarchical framework between the anomalous delusional beliefs, experiences and self-experiences (see figure 1). Therefore, in addition to perceptual anomalous experiences, metacognitive efficiency may predict occurrence of anomalous self-experiences. Dokic and Martin (2015) alluded to the idea that derealisation (anomalous self-experience) may be associated with metacognitive deficits. This study will empirically test this. Anomalous self-experiences have not been considered to be modality-specific, hence this has not been reflected in the construction of the scales, and will therefore not be assessed here.

[INSERT FIGURE 1 HERE]

In terms of the wider metacognitive model, metacognitive efficiency has been demonstrated as significantly impaired in psychosis compared to a healthy control group (Davies et al., in preparation), and as metacognition works in a hierarchical fashion, it may be expected that poor metacognitive efficiency in psychosis can impact the next component on the continuum: metacognitive experience, via metacognitive processes. However, Gilleen, Greenwood and David (2014) demonstrated that metacognitive experience is on average intact within a group of individuals with schizophrenia, whilst metacognitive knowledge, trait-like, stable beliefs about experience or abilities, is deficit. The dissociation between the different levels of metacognition may be the result of impaired metacognitive processes (self-reflection and set-shifting) (Gilleen, Greenwood & David, 2016), akin to various other studies (Engh et al., 2009; Medalia & Lim, 2004; Warman et al., 2006). It is currently unclear which metacognitive processes may be driving these metacognitive difficulties and limited studies have assessed metacognitive processing in young people with psychosis, which may provide knowledge regarding the nature of these metacognitive deficits. This study aims to further understand the difficulties in metacognitive monitoring in young people with psychosis. To test this, we will compare the metacognitive components and processes between young people with psychosis and matched healthy controls to identify key deficit components and then test the two models (figure 2 and 3) using regression analyses.

[INSERT FIGURE 2 HERE]

[INSERT FIGURE 3 HERE]

For peer review only - http://bmjopen.bmj.com/site/about/guidelines.xhtml

From this, we will assess which metacognitive components may impact on what individuals are doing day to day: their functional outcome. Functional outcome is a measurable aspect of an individual's specific activities of daily living. Research currently suggests functional outcome can be predicted by many factors including cognition, functional capacity (measured using real-life performance skills task, Patterson et al., 2001), symptoms particularly negative symptoms (Milev et al., 2005), which have been demonstrated to show a synergistic interaction with cognition to impact functioning (Greenwood et al, 2005), and, importantly here, metacognition. Metacognitive ability, measured using the Metacognition Assessment Interview (MAI) or MAS (Semerari et al., 2003; Semerari et al., 2012), appears to play a crucial role on functional outcome, independent of cognition and symptoms (James et al., 2016; Lysaker et al., 2011; Lysaker et al., 2014) and, in a recent study, Davies, Fowler & Greenwood (2017) demonstrated that metacognition partially mediates the relationship between cognition and functional capacity, and fully mediates the relationship between functional capacity and functional outcome. However, limited research has assessed whether metacognitive processes are also relevant in maintaining poor functional outcome over time from FEP, independent of cognition. With this in mind, a metacognitive factor will be derived from MAI and key metacognitive processes above to explore the impact on functional outcome (see figure 4). A supplementary longitudinal model will also explore the separate relationships, proposed to be evident cross-sectionally, can be demonstrated over a longer period of time from the first episode psychosis.

[INSERT FIGURE 4 HERE]

Functional outcome is measured using work status and specific functioning measures; assessing a variety of aspects such as personal care, interpersonal skills, community activities, work skills (Bowie et al., 2008). It has been associated with functional capacity in psychosis (Bowie et al., 2006), which itself is considered the most important predictor of real-world functional outcome (Green, 1996; Green et al., 2000) Alongside this, metacognition has been associated with functional capacity (specifically, comprehension and planning) after controlling for symptoms and neurocognition (Patterson et al., 2001). This may suggest that difficulties in mastery may result in an individual failing to apply their skills within real-life everyday activities, resulting in poor functional capacity, and subsequent poor functional outcome as functional capacity was shown to act as a mediator between neurocognition and functional outcome in schizophrenia (Bowie et al., 2006) and FEP (Davies, Fowler & Greenwood, 2017).

For peer review only - http://bmjopen.bmj.com/site/about/guidelines.xhtml

Functional outcome has also been associated with subjective recovery outcome (self-rated outcome reflecting sense of wellbeing and quality of life) in individuals with severe mental health difficulties (Malla & Payne, 2005; Provencher et al., 2002). However, these two outcome variables can be distinct as Bellack (2006) suggested functional outcome focuses on the medical model, whilst subjective outcome focuses on a consumer-focused recovery model. Kukla, Lysaker and Salyers (2013) demonstrated, within a schizophrenia sample, that metacognitive capabilities reflecting self-reflectivity and decentration were related to components of recovery beyond the effects of psychiatric symptoms, including aspects of quality of life (Phalen, Viswanadhan, Lysaker, Warman, 2015; Lysaker et al., 2005). The research above highlights the complex relationships between metacognition, functional capacity, and functional outcome. There is clear interest in the early identification of those at risk of poor outcome (objective and subjective), to target interventions to reduce this disability. This study will assess both objective and subjective function to allow a more in-depth understanding of functional recovery and the variables which are associated with this.

Collating the research above, it is evident that metacognition is a complex cognitive process which may work across a continuum with distinct metacognitive components, which interact via metacognitive processes. Although research has individually demonstrated deficits in various metacognitive components, no study has systematically tested the relationship between these components.

This study aims to develop and test a series of dynamic models to understand (i) the nature of deficits in metacognition compared to healthy controls (ii) the relationship between metacognitive components (iii) and the influences of metacognition on unusual experiences and objective/subjective functional outcome for young people with psychosis. If these proposed models can be demonstrated empirically, this can help us to further understand and remediate poor outcome within young people with psychosis.

Hypotheses

Hypothesis 1: Anomalous experiences will be associated with increased perceptual biases and poor metacognitive efficiency, and this relationship will be domain-specific.

Hypothesis 2: Metacognitive control processes will significantly predict metacognitive knowledge and metacognitive monitoring processes will significantly predict metacognitive experience. Metacognitive control processes will significantly predict metacognitive

For peer review only - http://bmjopen.bmj.com/site/about/guidelines.xhtml

experience and metacognitive monitoring processes will significantly predict metacognitive efficiency.

Hypothesis 3: Metacognitive variables (metacognitive knowledge, metacognitive processes and metacognitive experience) will significantly predict outcome measures (functional capacity, functional outcome and subjective recovery outcome) in young people with and without psychosis, even after controlling for symptoms and IQ.

Hypothesis 4: Metacognitive variables (metacognitive knowledge, metacognitive knowledge processes and metacognitive experience) will significantly predict outcome measures (functional capacity and functional outcome) at 36-month longitudinal follow-up of participants in young people with psychosis, even after controlling for symptoms and IQ.

METHODS

Design: A cross-sectional study will explore (i) deficits in metacognition between young people with psychosis and healthy controls; (ii) interrelations between different metacognitive components and processes across the whole sample and (iii) the contribution of specific novel metacognitive variables to unusual experiences and outcomes, and (iv) a longitudinal study will identify whether metacognition predicts experiences and outcomes at 36-month follow-up period (psychosis sample only).

Participants:

Seventy-three young people with psychosis will be recruited from first episode services and from a previous first episode psychosis (FEP) sample (n=80). Participants from the previous FEP study have baseline data on metacognition, functional capacity, functional outcome, symptoms and cognition (Davies, Fowler, & Greenwood, 2017). This data will form the baseline data for the longitudinal analysis. All participants with psychosis will be 18 – 40 years of age, able to read and communicate in English and receiving treatment for psychosis with a UK Early Intervention in Psychosis services at first assessment. Participants with organic causes for psychosis will be excluded.

Seventy-three healthy control participants will be recruited as a comparison group. These participants will be 18-40 years of age, able to read and communicate in English and matched on age, gender and education with the psychosis sample. Participants with current mental health problems or history of psychosis will be excluded following screening questions. A

For peer review only - http://bmjopen.bmj.com/site/about/guidelines.xhtml

primary diagnoses of substance use disorder and hearing or sight problems which cannot be corrected will be exclusion criteria for all participants. Data collection will be undertaken between 10th March 2017 and 4th May 2018. The end date of the study is 18th September 2018.

Patient and Public Involvement

Patient and Public Involvement is primarily via the Psychosis Interest Group run by Service User and Carer Involvement Coordinator at the Sussex Partnership NHS Foundation Trust Research and Development department, and service users within the Psychosis Theme Group (PTG). The first author met with the PTG to consult on the development of this project, including the design, methods, and procedure of the project. The lived experience group have viewed all the measures, including the two main computer tasks (visual and auditory tasks), and provided extensive feedback which has been incorporated into this project. Study participants who consent to receiving the study results will receive these by post/email. The first author and the Psychosis Theme Group will continue to meet to consult on recruitment procedures, and on the plans for dissemination to service user groups following data analysis.

Cross-sectional measures:

Anomalous experience measures

Anomalous perceptual experiences: Multimodal Unusual Sensory Experiences Questionnaire (Mitchell et al., et al. 2017). This measures anomalous perceptual/sensory experiences with 6 subscales. Both the full scale and subscales have been demonstrated as possessing good reliability (auditory r=0.72, visual r=0.72), good validity between clinical and non-clinical group (Cohen's d = 0.96) and significance with all other anomalous experience scale, and internal consistency (auditory α=0.82, visual α=0.88). The auditory and visual subscales will be used for this study which each provide a combined score for presence and frequency of anomalous experiences.

Anomalous self-experiences: Cambridge depersonalisation scale (trait and state versions) (Sierra & Berrios, 2000). The trait version includes 29 items measuring frequency (Never to All the time) and duration (Few seconds to More than a week) of anomalous self-experiences over the last 6 months. It demonstrates high internal consistency (>0.6), good validity with other scales (r=0.8) and good reliability (α= 0.89) (Sierra & Berrios, 2000), and is useful for

For peer review only - http://bmjopen.bmj.com/site/about/guidelines.xhtml

assessing depersonalisation in a schizophrenia group (Perona-Garcelan et al., 2011). The state version includes 22 items measuring anomalous self-experiences within-the-moment on a scale of intensity from 0-100. Subscales of the CDS are ‘alienation from surroundings’, ‘anomalous subjective recall’, ‘emotional numbing’ and ‘anomalous body experience’.

Anomalous delusional beliefs: Schizotypal Symptom Inventory (Hodgekins et al., 2012). This measure assesses residual psychotic symptoms, providing a total score with separate subscales for paranoia, anomalous experience and social anxiety (Hodgekins et al., 2012). This present study will use the paranoia subscale as a measure of anomalous delusional beliefs. This scale demonstrates high internal consistency (non-clinical sample α=0.87 and clinical sample α=0.92), good validity with other scales (PANSS with total r = 0.59 and paranoia r=0.6) and good test-retest reliability (0.85 for total and 0.6-0.84 for subscales).

Metacognition

Metacognitive efficiency

This encompasses separate computer –based visual and auditory detection task. The critical task in both paradigms is to make two forced-choice binary judgments of whether a visual or auditory stimulus (dot or tone) was present or absent (first judgement) within a noisy picture or presentation of white noise, and whether this was associated with high confidence or low confidence in the first judgement (second judgement).

The first judgement can be used to calculate a score for perceptual sensitivity and perceptual bias. *Perceptual sensitivity* (*d’*): the ability to correct report whether the stimulus (dot/tone) was either present or absent. Signal detection theory (SDT) posits that detection making involves depicting whether certain waveforms called signals may or may not be embedded within background ‘noise’, using internal responses (Green & Swets, 1974). *Perceptual bias* (*B*) is the tendency to report one decision over the other, e.g. stating the stimuli was present when it was in-fact absent. The internal responses from perceptual sensitivity score is then compared to a decision criterion (c) so all evidence above criteria elicits a response of ‘present’ compared to below the criteria elicits the response of ‘absent’ (Maniscalco, Peters & Lau, 2016). The perceptual sensitivity score and the decision criterion can be fitted to an empirical Receiver Operating Characteristic (ROC) curve, enabling us to assess perceptual biases in responses, e.g. whether someone was more or less likely to report stimuli as being present based on a lower/higher decision criterion. A perceptual bias score below 0 suggests a

For peer review only - http://bmjopen.bmj.com/site/about/guidelines.xhtml

bias towards reporting absence and a score above 1 suggests a bias towards reported presence of stimuli, previously used by Bentall and Slade (1985). Score of 1 is an 'ideal operator' (Bentall & Slade, 1985).

The second judgement can be used to calculate a score for metacognitive sensitivity and metacognitive efficiency. *Metacognitive sensitivity* (meta-*d'*) is the ability to discriminate between correct and incorrect judgments. Alike to the SDT above, metacognitive sensitivity is based on assessment of sensitivity (how well confidence ratings discriminate correct from incorrect trials) and response bias (overall propensity for reporting high confidence). However, this score from the second judgement must take into account first judgment performance (Maniscalco & Lau, 2014). Meta-*d'* indicates the *d'* that would have been predicted to result in the confidence rating assuming the signal detection theory. Optimal metacognitive sensitivity is when perceptual sensitivity score is matched. Meta-*d'* greater or less than *d'* indicates metacognition is better or worse than *d'* (Morales, Lau & Fleming, 2017). *Metacognitive efficiency* is one's ability to discriminate between one's own correct or incorrect perceptual decision, whilst taking into account objective performance (Fleming & Lau, 2014). This is calculated as meta-*d'*/*d'*. Metacognitive efficiency (meta-*d'*/*d'*) was chosen as a more robust form of perceptual metacognition, over metacognitive sensitivity (meta-*d'*) which was previously used by researchers (Maniscalco & Lau, 2012).

Performance on the first judgment will be held constant throughout the task and across participants (using a 1-up-2-down staircase procedure) to ensure that metacognitive ability is measured independent of task performance and produces valid scores.

Studies including signal detection tasks typically involve a large number of trials (~400), to avoid statistical bias and large variance in scores (Barrett, Dienes, & Seth, 2013). However, following a pilot study, we reduced the trials to 200, whilst maintaining reliable data. To ensure the feasibility of conducting this study within a clinical population, who may present difficulty with attention and concentration (Silverman, 1964) the two computer tasks have been developed from a pilot study within a healthy student population (Wright, Fowler, & Greenwood, in preparation).

Alongside this, participants will be asked to prospectively and retrospectively rate their performance on the detection tasks. These ratings will be used to assess metacognitive knowledge and metacognitive experience.

For peer review only - http://bmjopen.bmj.com/site/about/guidelines.xhtml

Metacognitive knowledge: The prospective rating ("How well do you think you will perform overall? For instance, if you think you will get it right every time, select 100 (all correct). If you think you will be correct none of the time, select 0 (none correct). You can select any value in between zero and 100 to indicate what percentage you think you will correctly identify") will assess task-related metacognitive knowledge.

Metacognitive experience: The retrospective rating ("How well do you think you performed overall on the task? For instance, if you think you were right every time, select 100 (all correct). If you think you were correct none of the time, select 0 (none correct). You can select any value in between zero and 100 to indicate what percentage you think you correctly identified") will assess task-related metacognitive experience.

Metacognitive ability: This will be assessed using the Metacognitive Assessment Interview (Semerari et al., 2012), to measure metacognitive knowledge. This measure assesses the ability to understand "the self" and "the other"; termed as one multidimensional construct as 'metacognition'. This measure has demonstrated good inter-rater reliability and internal consistency (α=0.90 for total metacognition), factorial validity, and reliability (r=0.62 to 0.90) (Semerari et al., 2012).

Metacognitive processes (monitoring and control): Monitoring processes will be assessed using self-reflectivity subscale of Cognitive insight scale (Beck, Baruch, Balter, Steer, & Warman, 2004). This measure possesses good internal consistency (α=0.68), convergent validity (with SUMD-A delusions, r= -0.67) (Beck, Baruch, Balter, Steer, & Warman, 2004) and test-retest reliability (r=0.90) (Pedrelli et al., 2004). Studies have demonstrated this measure is appropriate to assess metacognitive monitoring (David et al., 2012; Gilleen, Greenwood & David, 2016). Control processes will be assessed via set-shifting using trail-making task (Reitan, 1971) part B-A. This measure possesses good internal consistency (TMT-A α=0.39, TMT-B α=0.71), convergent validity (with Task Switching Paradigm r=0.32) (Sanchez-Cubillo et al., 2009), and good reliability of other forms of TMT (r=0.78) (Franzen et al., 1996). This measure is appropriate to assess metacognitive control processes (Gilleen, Greenwood & David, 2016).

Function

Functional outcome: Time Use Survey (adapted from; Short, 2006) provides a retrospectively rated objective measure for hours spent engaging in structured activity per week (Fowler et

For peer review only - http://bmjopen.bmj.com/site/about/guidelines.xhtml

al., 2009). This measure has been used within a First Episode Psychosis sample to assess functioning (Hodgekins et al., 2015; Cella et al., 2016). This measure has good reliability (inter-rater reliability at 0.99; Hodgekins et al., 2015), coder reliability (89% accuracy, Short, 2003), good validity as differences in Time-Use have been demonstrated between different stages of psychosis, representing social recovery in psychosis (Hodgekins et al., 2015), validity as TUS is comparable to studies using functioning measures (Hodgekins et al., 2012a).

Functional capacity: The UCSD Performance-Based Skills Assessment (Patterson et al., 2001) provides a total score for real-life performance skills based on simulated tasks. It was adapted to be applicable for UK participants. This measure demonstrates high internal consistency (α=0.88), good validity with other scales (DAFS r=0.86) and good test-retest reliability (r=0.91) (Harvey, Velligan & Bellack, 2007; Mausbach et al., 2009).

Subjective recovery outcome: The Questionnaire of Process of Recovery (Neil et al., 2009) provides a score for an individual's subjective functioning (psychosis participants only). This scale has two subscales: intrapersonal items related to hope, empowerment, confidence, and interpersonal related to connectedness with others, others help/care, reliance. This possesses good internal consistency (intrapersonal subscale, α=.94, interpersonal subscale, α=.77), construct validity (GHQ total score: intra, r=-.83 inter, r=.52) and reliability (intra, r=.87, inter, r.77) (Neil et al., 2009).

Covariates

Symptom severity: This assesses symptoms of psychosis using Positive and Negative Syndrome Scale (Kay & Fiszbein, 1987) (clinical participants only), which is the mostly widely used standardised instrument for assessing symptom severity in schizophrenia (Hermes et al., 2012). This measure has demonstrated good reliability and validity, and appropriate inverse correlations between positive and negative subscales (Kay et al., 1988). This measure has good internal consistency (agreement for PANSS items r=0.69 – 0.94), construct validity (between PANSS and Andreasen rating system, r=0.77) and reliability (interrater correlations for PANSS scales ranged from 0.83 to 0.87) (Kay & Fiszbein, 1987).

Cognitive ability: This includes verbal IQ: Vocabulary task (Wechsler, 1999) is a measure of an individual's verbal knowledge and fund of information. This measure as good internal

For peer review only - http://bmjopen.bmj.com/site/about/guidelines.xhtml

consistency (correlation with other cognitive measures range r=0.54 to 0.79), construct validity (with WASI-III, r=0.88) and reliability (0.90 to 0.89) and test-retest (0.88) (Wechsler, 1999); and performance IQ: Matrix reasoning task (Wechsler, 1999) is a measure of individual's ability to mentally manipulate abstract symbols and perceive the relationship among them. This measure as good internal consistency (correlation with other cognitive measures range r=0.59 to 0.63), construct validity (with WASI-III, r=0.66) and reliability (0.88 to 0.96) and test-retest (0.76) (Wechsler, 1999).

Longitudinal measures:

For participants who have baseline data, this comprises of following measures outlined above: metacognitive knowledge and metacognitive monitoring processes; functional outcome and functional capacity; and all covariates.

ANALYSIS

Sample size: Two sample size estimates have been combined to ensure the analyses have sufficient power to detect effects.

Firstly, sample size to detect differences between individuals with psychosis and healthy control participants on metacognitive efficiency. Using G power with .8 power, .57 effect size based on a previous metacognitive efficiency task in psychosis (Davies et al. in preparation) and 0.05 alpha, the proposed total sample size is 28 (14 per group). Secondly, a regression analysis to assess the predictive value of metacognitive knowledge, processes and experience on outcome measures (controlling for symptoms and IQ) will be conducted. G power estimation was used for a power calculation based on a power of .80, effect size of .313 (Davies, Fowler & Greenwood, 2017) and alpha of .05. This suggested for 6 predictors a total of 55 participants are required.

In terms of the mediation analysis, power estimation was calculated using the monte carlo method to estimate power for complex mediation models (see Thoemmes, MacKinnon & Resier, 2010). Using fixed parameters from Davies, Fowler and Greenwood (2017) and power at .8, this suggested a total sample size of 146 participants to detect mediation effects, outlined in the above model.

In terms of the longitudinal analysis, as many of the original sample (n=80) as possible will be followed up to maximise statistical power.

For peer review only - http://bmjopen.bmj.com/site/about/guidelines.xhtml

Planned data analysis: Data will be double entered and checked for accuracy, and checked for outliers.

Missing data will be considered as 'Missing at random' (MAR), which means the missing variables are related to additional observed variables within the data, but values of missing data itself. Missing data will be treated according to best practice (McKnight et al., 2007). Principled missing data methods will be used which combine available information from the observed data to estimate the population parameters and/or the missing data mechanism (Dong and Peng, 2013). Full information maximum likelihood (FIML) involves using all the observed data and creating values for missing data using maximum likelihood estimations. This works well provided that the model for the complete data is realistic (Schafer & Graham, 2002). FIML will be used within this study which is considered most appropriate for MAR data and for mediation analyses (Allison, 2003). Quantitative data (including demographic information) will be reported using descriptive statistics, e.g. means and standard deviations.

Data will be analysed using SPSS and Mplus software. Group differences of all metacognitive measures and perceptual biases between young people with psychosis and healthy controls will be assessed, controlling for age and IQ. Linear regression analyses will be used to assess how metacognition (and perceptual biases) predicts anomalous experiences. Cross-sectional and longitudinal predictive analyses will be conducted, including regression analyses which will assess whether metacognitive components; knowledge, experience, efficiency are predicted by metacognitive processes. A mediation analysis will be used to whether functional capacity, functional outcome and subjective recovery outcome are predicted by metacognition and metacognitive processes, whilst controlling for symptoms and cognition. These analyses will include bootstrapped bias-corrected confidence intervals.

DISCUSSION

Our assumption is that metacognitive variables predict, and maintain, anomalous events, poor objective and subjective recovery outcome in young people with and without psychosis. Particularly in young people with psychosis, metacognitive deficits may predict long-term functional outcome.

The study results will be an important addition to the literature and for clinicians for four main reasons: i) this study tests a proposed model from previous literature which may help understand poor functional outcome in psychosis, ii) from this, novel intervention studies can

For peer review only - http://bmjopen.bmj.com/site/about/guidelines.xhtml

be developed to tackle the potential metacognitive deficits in psychosis which predict this poor functional outcome, iii) this is one of the first studies to assess metacognition as a longitudinal predictor of functional outcome in young people with psychosis sample, and finally iv) our studies use up-to-date paradigms within the field of metacognition to avoid biases.

ETHICS AND DISSEMINATION

This study has been reviewed and approved by Camberwell St. Giles Research Ethics Committee (reference number: 17/LO/0055). The data will be stored securely in accordance with usual NHS procedures and data will be governed by the sponsor: University of Sussex. This research project will be reported within a PhD thesis, and will be written up for publication in scientific journals.

REFERENCES

Allison, P. D. (2003). Missing data techniques for structural equation modeling. *Journal of abnormal psychology*, *112*(4), 545.

Balzan, R. P. (2016). Overconfidence in psychosis: The foundation of delusional conviction?. *Cogent Psychology*, *3*(1), 1135855.

Barrett, A. B., Dienes, Z., & Seth, A. K. (2013). Measures of metacognition on signal-detection theoretic models. *Psychological Methods*, *18*, 535–52. http://doi.org/10.1037/a0033268

Beck, A. T., Baruch, E., Balter, J. M., Steer, R. a., & Warman, D. M. (2004). A new instrument for measuring insight: The Beck Cognitive Insight Scale. *Schizophrenia Research*, *68*, 319–329. doi:10.1016/S0920-9964(03)00189-0

Bell, V., Halligan, P. W., & Ellis, H. D. (2006). The Cardiff Anomalous Perceptions Scale (CAPS): A New Validated Measure of Anomalous Perceptual Experience. *Schizophrenia Bulletin*, *32*(2), 366–377. doi:10.1093/schbul/sbj014

Bellack, A. S. (2006). Scientific and consumer models of recovery in schizophrenia: concordance, contrasts, and implications.

Bentall, R. P., & Slade, P. D. (1985). Reality testing and auditory hallucinations: a signal detection analysis. *British Journal of Clinical Psychology*, *24*(3), 159-169.

For peer review only - http://bmjopen.bmj.com/site/about/guidelines.xhtml

Bowie, C. R., Reichenberg, A., Patterson, T. L., Heaton, R. K., & Harvey, P. D. (2006). Determinants of real-world functional performance in schizophrenia subjects: correlations with cognition, functional capacity, and symptoms. *American Journal of Psychiatry*, *163*(3), 418-425.

Bowie, C. R., Leung, W. W., Reichenberg, A., McClure, M. M., Patterson, T. L., Heaton, R. K., & Harvey, P. D. (2008). Predicting schizophrenia patients' real-world behavior with specific neuropsychological and functional capacity measures. *Biological psychiatry*, *63*(5), 505-511.

Brett, C. M. C., Johns, L. C., Peters, E. P., & McGuire, P. K. (2009). The role of metacognitive beliefs in determining the impact of anomalous experiences: a comparison of help-seeking and non-help-seeking groups of people experiencing psychotic-like anomalies. *Psychological Medicine*, *39*(6), 939–950. doi:10.1017/S0033291708004650

Cella, M., Edwards, C., & Wykes, T. (2016). A question of time: A study of time use in people with schizophrenia. *Schizophrenia research*, *176*(2), 480-484.

David, A. S., Bedford, N., Wiffen, B., & Gilleen, J. (2012). Failures of metacognition and lack of insight in neuropsychiatric disorders. *Phil. Trans. R. Soc. B*, *367*(1594), 1379-1390.

Davies, G., Fowler, D., & Greenwood, K. (2017). Metacognition as a Mediating Variable Between Neurocognition and Functional Outcome in First Episode Psychosis. *Schizophrenia Bulletin*, sbw128.

Davies, G., Rae, C., Garfinkel, S., Seth, A., Medford, N., Critchley, H., & Greenwood, K. (In preparation). Impairment of perceptual metacognitive accuracy and reduced prefrontal grey matter volume in First-Episode Psychosis.

Dokic, J., & Martin, J. R. (2015). Felt Reality and the Opacity of Perception. *Topoi*. doi:10.1007/s11245-015-9327-2

Dong, Y., & Peng, C. Y. J. (2013). Principled missing data methods for researchers. *SpringerPlus*, *2*(1), 222.

Efklides, A. (2008). Metacognition: Defining its facets and levels of functioning in relation to self-regulation and co-regulation. *European Psychologist*, *13*(4), 277-287.

For peer review only - http://bmjopen.bmj.com/site/about/guidelines.xhtml

Eisenacher, S., Rausch, F., Ainser, F., Mier, D., Veckenstedt, R., Schirmbeck, F., & Lewien, A. (2015). Investigation of metamemory functioning in the at-risk mental state for psychosis, 3329–3340. http://doi.org/10.1017/S0033291715001373

Engh, J. A., Friis, S., Birkenaes, A. B., Jónsdóttir, H., Klungsøyr, O., Ringen, P. A., ... & Andreassen, O. A. (2009). Delusions are associated with poor cognitive insight in schizophrenia. *Schizophrenia bulletin*, sbn193.

Fleming, S. M., Ryu, J., Golfinos, J. G., & Blackmon, K. E. (2014). Domain-specific impairment in metacognitive accuracy following anterior prefrontal lesions. *Brain*, *137*(10), 2811-2822.

Fleming, S. M., & Lau, H. C. (2014). How to measure metacognition. *Frontiers in human neuroscience*, *8*, 443.

Fletcher, P. C., & Frith, C. D. (2009). Perceiving is believing: a Bayesian approach to explaining the positive symptoms of schizophrenia. *Nature Reviews Neuroscience*, *10*(1), 48-58.

Fowler, D., Hodgekins, J., Howells, L., Millward, M., Ivins, A., Taylor, G., ... & Macmillan, I. (2009). Can targeted early intervention improve functional recovery in psychosis? A historical control evaluation of the effectiveness of different models of early intervention service provision in Norfolk 1998–2007. *Early Intervention in Psychiatry*, *3*(4), 282-288.

Franzen, M. D., Paul, D., & Iverson, G. L. (1996). Reliability of alternate forms of the trail making test. *The Clinical Neuropsychologist*, *10*(2), 125-129.

Freeman, D., Garety, P. A., Kuipers, E., Fowler, D., & Bebbington, P. E. (2002). A cognitive model of persecutory delusions. *British Journal of Clinical Psychology*, *41*(4), 331-347.

Garety, P. A., Kuipers, E., Fowler, D., Freeman, D., & Bebbington, P. E. (2001). A cognitive model of the positive symptoms of psychosis. *Psychological medicine*, *31*(2), 189-195.

Gawęda, Ł., Woodward, T. S., Moritz, S., & Kokoszka, A. (2013). Impaired action self-monitoring in schizophrenia patients with auditory hallucinations. *Schizophrenia research*, *144*(1), 72-79.

For peer review only - http://bmjopen.bmj.com/site/about/guidelines.xhtml

Green, D. M., & Swets, J. A. (1974). Signal detection theory and psychophysics (Rev. ed.). *Huntington, NY: RF Krieger*.

Green, M. F. (1996). What are the functional consequences of neurocognitive deficits in schizophrenia?. *The American journal of psychiatry*, *153*(3), 321.

Green, M. F., Kern, R. S., Braff, D. L., & Mintz, J. (2000). Neurocognitive deficits and functional outcome in schizophrenia: are we measuring the" right stuff"?.

Greenwood, K. E., Landau, S., & Wykes, T. (2005). Negative symptoms and specific cognitive impairments as combined targets for improved functional outcome within cognitive remediation therapy. *Schizophrenia Bulletin*, *31*(4), 910-921.

Gilleen, J., Greenwood, K., & David, A. S. (2014). The role of memory in awareness of memory deficits in Alzheimer's disease, schizophrenia, and brain injury. *Journal of clinical and experimental neuropsychology*, *36*(1), 43-57.

Gilleen, J., David, A., & Greenwood, K. (2016). Self-reflection and set-shifting mediate awareness in cognitively preserved schizophrenia patients. *Cognitive neuropsychiatry*, *21*(3), 185-196.

Harvey, P. D., Velligan, D. I., & Bellack, A. S. (2007). Performance-based measures of functional skills: usefulness in clinical treatment studies. *Schizophrenia bulletin*, *33*(5), 1138-1148.

Hermes, E. D., Sokoloff, D. M., Stroup, T. S., & Rosenheck, R. A. (2012). Minimum clinically important difference in the Positive and Negative Syndrome Scale using data from the CATIE Schizophrenia Trial. *The Journal of clinical psychiatry*, *73*(4), 526.

Hodgekins, J., Coker, S., Freeman, D., Ray-Glover, K., Bebbington, P., Garety, P…. Fowler, D. (2012). Assessing Levels of Subthreshold Psychotic Symptoms in the Recovery Phase: the Schizotypal Symptoms Inventory (SSI). *Journal of Experimental Psychopathology*. Doi: 10.5127/jep.021211.

Hodgekins, J. (2012a). *Investigating the psychological typology of social recovery in individuals with first episode psychosis* (Doctoral dissertation, University of East Anglia).

For peer review only - http://bmjopen.bmj.com/site/about/guidelines.xhtml

Hodgekins, J., Birchwood, M., Christopher, R., Marshall, M., Coker, S., Everard, L., … Fowler, D. (2015). Investigating trajectories of social recovery in individuals with first-episode psychosis: A latent class growth analysis. *British Journal of Psychiatry*, *207*(6), 536–543. http://doi.org/10.1192/bjp.bp.114.153486

James, A. V., Hasson-Ohayon, I., Vohs, J., Minor, K. S., Leonhardt, B. L., Buck, K. D., ... & Lysaker, P. H. (2016). Metacognition moderates the relationship between dysfunctional self-appraisal and social functioning in prolonged schizophrenia independent of psychopathology. *Comprehensive Psychiatry*, *69*, 62-70.

Kay SR, & Fiszbein A, O. L. (1987). The Positive and Negative Syndrome Scale for schizophrenia. Schizophr Bull., 13(2), 261–276. doi:10.1093/schbul/13.2.261

Kay, S. R., Opler, L. A., & Lindenmayer, J. P. (1988). Reliability and validity of the positive and negative syndrome scale for schizophrenics. *Psychiatry Research*, *23*(1), 99–110. http://doi.org/10.1016/0165-1781(88)90038-8

Kelleher, I., Connor, D., Clarke, M. C., Devlin, N., Harley, M., & Cannon, M. (2012). Prevalence of psychotic symptoms in childhood and adolescence: a systematic review and meta-analysis of population-based studies. *Psychological Medicine*, *42*(09), 1857–1863. doi:10.1017/S0033291711002960

Kircher, T. T., Koch, K., Stottmeister, F., & Durst, V. (2007). Metacognition and reflexivity in patients with schizophrenia. *Psychopathology*, *40*(4), 254-260.

Kok, Kouider, Lange, & Supe. (2015). Spontaneous Activity Patterns in Primary Visual Cortex, *35*(37), 12947–12953.

Kukla, M., Lysaker, P. H., & Salyers, M. P. (2013). Do persons with schizophrenia who have better metacognitive capacity also have a stronger subjective experience of recovery?. *Psychiatry Research*, *209*(3), 381-385.

Lower, R., Wilson, J., Medin, E., Corlett, E., Turner, R., Wheeler, K., & Fowler, D. (2015). Evaluating an early intervention in psychosis service for 'high-risk'adolescents: symptomatic and social recovery outcomes. *Early intervention in psychiatry*, *9*(3), 260-267.

Lysaker, P. H., Carcione, A., Dimaggio, G., Johannesen, J. K., Nicolò, G., Procacci, M., & Semerari, A. (2005). Metacognition amidst narratives of self and illness in

For peer review only - http://bmjopen.bmj.com/site/about/guidelines.xhtml

schizophrenia: associations with neurocognition, symptoms, insight and quality of life. *Acta psychiatrica scandinavica*, *112*(1), 64-71.

Lysaker, P. H., McCormick, B. P., Snethen, G., Buck, K. D., Hamm, J. A., Grant, M., ... & Dimaggio, G. (2011). Metacognition and social function in schizophrenia: associations of mastery with functional skills competence. *Schizophrenia research*, *131*(1), 214-218.

Lysaker, P. H., Leonhardt, B. L., Pijnenborg, M., van Donkersgoed, R., de Jong, S., & Dimaggio, G. (2014). Metacognition in schizophrenia spectrum disorders: methods of assessment and associations with neurocognition, symptoms, cognitive style and function. *Isr J Psychiatry Relat Sci*, *51*(1), 54-61.

Malla, A., & Payne, J. (2005). First-Episode Psychosis : Psychopathology , Quality of Life , and Functional Outcome, *31*(3), 650–671. http://doi.org/10.1093/schbul/sbi031

Maniscalco, B., Peters, M. A., & Lau, H. (2016). Heuristic use of perceptual evidence leads to dissociation between performance and metacognitive sensitivity. *Attention, Perception, & Psychophysics*, *78*(3), 923-937.

Maniscalco, B., & Lau, H. (2014). Signal detection theory analysis of type 1 and type 2 data: meta-d′, response-specific meta-d′, and the unequal variance SDT model. In *The cognitive neuroscience of metacognition* (pp. 25-66). Springer Berlin Heidelberg.

Maniscalco, B., & Lau, H. (2012). A signal detection theoretic approach for estimating metacognitive sensitivity from confidence ratings. *Consciousness and cognition*, *21*(1), 422-430.

Mausbach, B. T., Moore, R., Bowie, C., & Cardenas, V. (2009). A Review of Instruments for Measuring Functional Recovery in Those Diagnosed With Psychosis, *35*(2), 307–318. http://doi.org/10.1093/schbul/sbn152

McKnight, P. E., McKnight, K. M., Sidani, S., & Figueredo, A. J. (2007). *Missing data: A gentle introduction*. Guilford Press.

Medalia, A., & Lim, R. W. (2004). Self-awareness of cognitive functioning in schizophrenia. *Schizophrenia research*, *71*(2), 331-338.

Milev, P., Ho, B. C., Arndt, S., & Andreasen, N. C. (2005). Predictive values of neurocognition and negative symptoms on functional outcome in schizophrenia: a

For peer review only - http://bmjopen.bmj.com/site/about/guidelines.xhtml

longitudinal first-episode study with 7-year follow-up. *American Journal of Psychiatry*, *162*(3), 495-506.

Mintz, S., & Alpert, M. (1972). Imagery vividness, reality testing, and schizophrenic hallucinations. *Journal of Abnormal Psychology*, *79*(3), 310.

Mitchell, C. A., Maybery, M. T., Russell-Smith, S. N., Collerton, D., Gignac, G. E., & Waters, F. (2017). The Structure and Measurement of Unusual Sensory Experiences in Different Modalities: The Multi-Modality Unusual Sensory Experiences Questionnaire (MUSEQ). *Frontiers in Psychology*, *8*, 1363.

Morales, J., Lau, H., & Fleming, S. M. (2017). Domain-Specific Patterns of Activity Support Metacognition in Human Prefrontal Cortex. *bioRxiv*, 172445.

Moritz, S., Woodward, T. S., Whitman, J. C., & Cuttler, C. (2005). Confidence in errors as a possible basis for delusions in schizophrenia. *The Journal of nervous and mental disease*, *193*(1), 9-16.

Moritz, S., Ramdani, N., Klass, H., Andreou, C., Jungclaussen, D., Eifler, S., … Zink, M. (2014). Overconfidence in incorrect perceptual judgments in patients with schizophrenia. *Schizophrenia Research: Cognition*, *1*(4), 165–170. http://doi.org/10.1016/j.scog.2014.09.003

Mussgay, L., & Hertwig, R. (1990). Signal detection indices in schizophrenics on a visual, auditory, and bimodal continuous performance test. *Schizophrenia research*, *3*(5), 303-310.

Neil, S. T., Kilbride, M., Pitt, L., Nothard, S., Welford, M., Sellwood, W., & Morrison, A. P. (2009). The questionnaire about the process of recovery (QPR): a measurement tool developed in collaboration with service users. *Psychosis*, *1*(2), 145-155.

Nelson, T. O., & Narens, L. (1990). The psychology of learning and motivation. *Metamemory: A theoretical framework and new findings*.

Nelson, T.O. (1984) A comparison of current measures of the accuracy of feeling-of-knowing predictions. Psychological Bulletin, 95(1), 109–133.

Nelson, B., Parnas, J., & Sass, L. A. (2014). Disturbance of minimal self (ipseity) in schizophrenia: clarification and current status.

For peer review only - http://bmjopen.bmj.com/site/about/guidelines.xhtml

Patterson, T. L., Goldman, S., McKibbin, C. L., Hughs, T., & Jeste, D. V. (2001). UCSD Performance-Based Skills Assessment: development of a new measure of everyday functioning for severely mentally ill adults. *Schizophrenia bulletin*, *27*(2), 235-245.

Pedrelli, P., McQuaid, J. R., Granholm, E., Patterson, T. L., McClure, F., Beck, A. T., & Jeste, D. V. (2004). Measuring cognitive insight in middle-aged and older patients with psychotic disorders. *Schizophrenia research*, *71*(2), 297-305.

Perona-Garcelán, S., Carrascoso-López, F., García-Montes, J. M., Vallina-Fernández, O., Pérez-Álvarez, M., Ductor-Recuerda, M. J., ... & Gómez-Gómez, M. T. (2011). Depersonalization as a mediator in the relationship between self-focused attention and auditory hallucinations. *Journal of Trauma & Dissociation*, *12*(5), 535-548.

Phalen, P. L., Viswanadhan, K., Lysaker, P. H., & Warman, D. M. (2015). The relationship between cognitive insight and quality of life in schizophrenia spectrum disorders: Symptom severity as potential moderator. *Psychiatry research*, *230*(3), 839-845.

Provencher, H. L., Gregg, R., Mead, S., & Mueser, K. T. (2002). The role of work in the recovery of persons with psychiatric disabilities. *Psychiatric rehabilitation journal*, *26*(2), 132.

Reininghaus, U., Kempton, M., Valmaggia, L. R., Craig, T., Garety, P., Onyejiaka, A., & Gayer-Anderson, C. (2016). Stress sensitivity, aberrant salience , and threat anticipation in early psychosis : an experience sampling study. doi:10.1093/schbul/sbv190

Reitan, R. M. (1971). Trail making test results for normal and brain-damaged children. *Perceptual and motor skills*, *33*(2), 575-581.

Sanchez-Cubillo, I., Perianez, J. A., Adrover-Roig, D., Rodriguez-Sanchez, J. M., Rios-Lago, M., Tirapu, J. E. E. A., & Barcelo, F. (2009). Construct validity of the Trail Making Test: role of task-switching, working memory, inhibition/interference control, and visuomotor abilities. *Journal of the International Neuropsychological Society*, *15*(3), 438-450.

Schafer, J. L., & Graham, J. W. (2002). Missing data: our view of the state of the art. *Psychological methods*, *7*(2), 147.Semerari, A., Carcione, A., Dimaggio, G., Falcone, M., Nicolo, G., Procacci, M., & Alleva, G. (2003). How to evaluate

For peer review only - http://bmjopen.bmj.com/site/about/guidelines.xhtml

metacognitive functioning in psychotherapy? The metacognition assessment scale and its applications. *Clinical Psychology & Psychotherapy*, *10*(4), 238-261.

Semerari, A., Carcione, A., Dimaggio, G., Falcone, M., Nicolò, G., Procacci, M., & Alleva, G. (2003). How to evaluate metacognitive functioning in psychotherapy? The metacognition assessment scale and its applications. *Clinical Psychology & Psychotherapy*, *10*(4), 238-261.

Semerari, A., Cucchi, M., Dimaggio, G., Cavadini, D., Carcione, A., Battelli, V., ... & Ronchi, P. (2012). The development of the Metacognition Assessment interview: instrument description, factor structure and reliability in a non-clinical sample. *Psychiatry Research*, *200*(2), 890-895.

Shea, N., Boldt, A., Bang, D., Yeung, N., Heyes, C., & Frith, C. D. (2014). Supra-personal cognitive control and metacognition. *Trends in Cognitive Sciences*, *18*(4), 186–193. doi:10.1016/j.tics.2014.01.006

Sherman, M., Barrett, A., and Kanai, R. (2015) *Inferences about consciousness using subjective reports of confidence.* In: Overgaard, Morten (ed.) Behavioral Methods in Consciousness Research. Oxford University Press, Oxford, UK. ISBN 9780199688890.

Sherman, M T, Seth, A K, Barrett, A B and Kanai, R (2015) *Prior expectations facilitate metacognition for perceptual decision.* Consciousness and Cognition, 35. pp. 53-65. ISSN 1053-8100.

Short, S. (2003). The United Kingdom 2000 Time Use Survey Technical Report. *Office for National Statistics.*

Short, S. (2006). Review of the UK 2000 Time Use Survey. *Office for National Statistics*, *11*.

Sierra, M., & Berrios, G. E. (2000). The Cambridge Depersonalisation Scale: A new instrument for the measurement of depersonalisation. *Psychiatry research*, *93*(2), 153-164.

Silverman, J. (1964). The problem of attention in research and theory in schizophrenia. *Psychological Review*, *71*(5), 352.

Teufel, C., Kingdon, A., Ingram, J. N., Wolpert, D. M., & Fletcher, P. C. (2010). Deficits in sensory prediction are related to delusional ideation in healthy individuals. *Neuropsychologia*, *48*(14), 4169-4172.

For peer review only - http://bmjopen.bmj.com/site/about/guidelines.xhtml

Teufel, C., Subramaniam, N., Dobler, V., Perez, J., Finnemann, J., Mehta, P. R., ... & Fletcher, P. C. (2015). Shift toward prior knowledge confers a perceptual advantage in early psychosis and psychosis-prone healthy individuals. *Proceedings of the National Academy of Sciences*, *112*(43), 13401-13406.

Thoemmes, F., MacKinnon, D. P., & Reiser, M. R. (2010). Power analysis for complex mediational designs using Monte Carlo methods. *Structural Equation Modeling*, *17*(3), 510-534.

Warman, D. M., & Martin, J. M. (2006). Cognitive insight and delusion proneness: an investigation using the Beck Cognitive Insight Scale. *Schizophrenia research*, *84*(2), 297-304.

Warman, D. M., Lysaker, P. H., & Martin, J. M. (2007). Cognitive insight and psychotic disorder: The impact of active delusions. *Schizophrenia Research*, *90*(1–3), 325–333. http://doi.org/10.1016/j.schres.2006.09.011. Warman, D. M. (2008). Reasoning and delusion proneness: confidence in decisions. *The Journal of nervous and mental disease*, *196*(1), 9-15.

Wechsler, D. (1999). Wechsler Abbreviated Scale of Intelligence™ (WASI™).

Wright, A.C., Fowler, D., & Greenwood, K. (in preparation). Understanding the association between anomalous experiences and perceptual and metacognitive ability within the general population: a pilot study.

Wells, A., & Purdon, C. (1999). Metacognition and cognitive-behaviour therapy: a special issue. *Clinical Psychology & Psychotherapy*, *6*(2), 71-72.

AUTHOR’S CONTRIBUTIONS

Abigail Wright designed the study, supervised by Dr Kathryn Greenwood and Professor David Fowler. We thank Dr Samuel Berens who assisted in the design and coding of the Matlab tasks. We thank the Patient and Public Involvement forum, Psychosis Theme Group (PTG), who reviewed and commented on the project, in order to provide a lived experience perspective. The Sussex Psychosis Research Interest Group and Sackler Centre for Consciousness Science have also reviewed the original protocol.

For peer review only - http://bmjopen.bmj.com/site/about/guidelines.xhtml

FUNDING STATEMENT

This work was supported by Sussex Partnership NHS Foundation Trust and Economic Social Research Council, through a PhD studentship awarded to the first author (Reference: ES/J500173/1).

CONFLICTS OF INTERESTS STATEMENT

There is no conflict of interest.

Figure legends

Figure 1: Proposed model including associations between perceptual bias and metacognitive efficiency with various anomalous events: anomalous experiences and beliefs, and the associations between metacognitive aspects.

Figure 2: Part of figure 1 model. Proposed associations between metacognitive efficiency and metacognitive experience with monitoring and controlling processes.

Figure 3: Part of figure 1 model. Proposed associations between metacognitive experience and metacognitive knowledge with monitoring and controlling processes.

Figure 4: Proposed model for cross-sectional and longitudinal analyses including indirect effects from symptoms and neurocognition to metacognition and functional capacity to functional and subjective outcome.

For peer review only

For peer review only - http://bmjopen.bmj.com/site/about/guidelines.xhtml

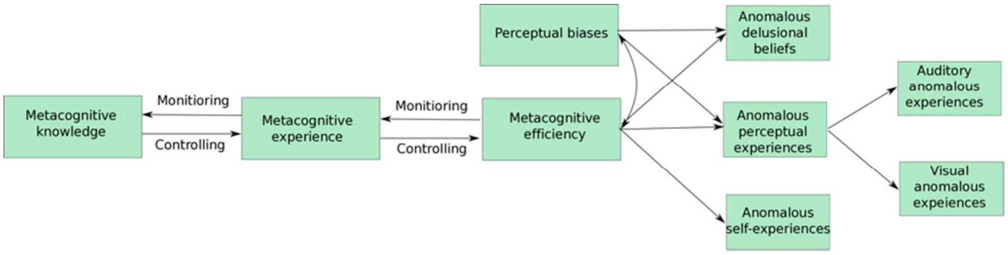

Figure 1: Proposed model including associations between perceptual bias and metacognitive efficiency with various anomalous events: anomalous experiences and beliefs, and the associations between metacognitive aspects.

For peer review only

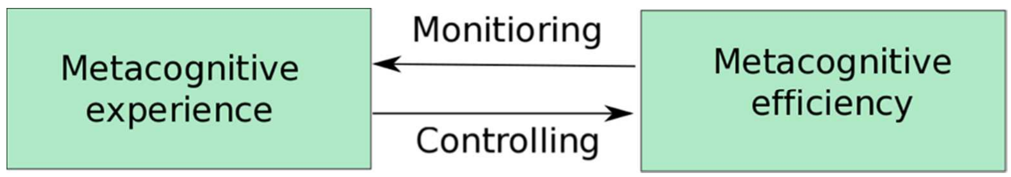

Figure 2: Part of figure 1 model. Proposed associations between metacognitive efficiency and metacognitive experience with monitoring and controlling processes.

For peer review only

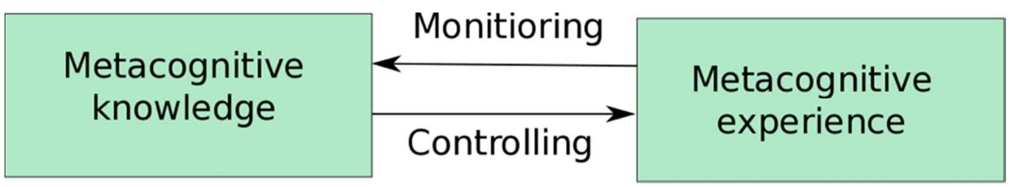

Figure 3: Part of figure 1 model. Proposed associations between metacognitive experience and metacognitive knowledge with monitoring and controlling processes.

For peer review only

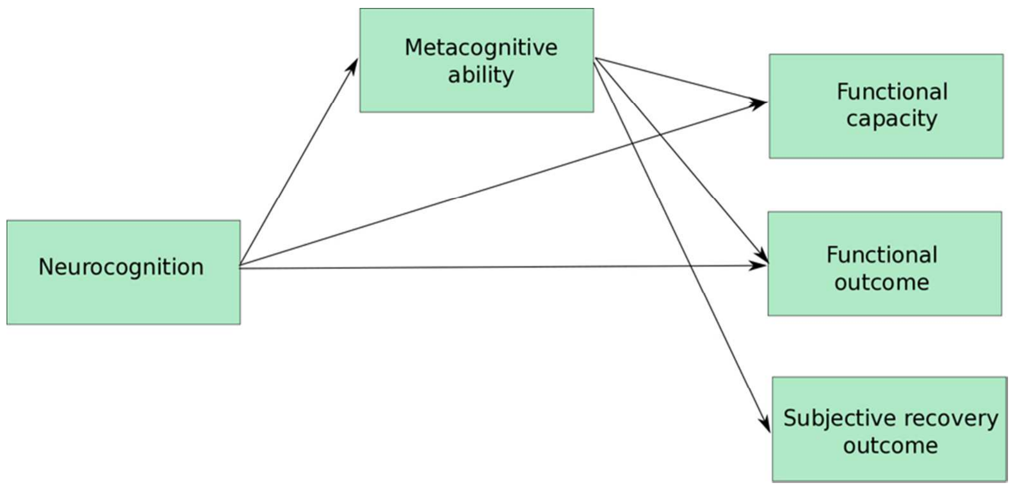

Figure 4: Proposed model for cross-sectional and longitudinal analyses including indirect effects from symptoms and neurocognition to metacognition and functional capacity to functional and subjective outcome.

For peer review only

BMJ Open

Developing a dynamic model of anomalous experiences and function in young people with or without psychosis: a cross-sectional and longitudinal study protocol.

Journal:	*BMJ Open*
Manuscript ID	bmjopen-2018-022546.R1
Article Type:	Protocol
Date Submitted by the Author:	06-Jul-2018
Complete List of Authors:	Wright, Abigail; University of Sussex, School of Psychology<br>Fowler, David; University of Sussex, School of Psychology<br>Greenwood, Kathryn; University of Sussex, School of Psychology; Sussex Partnership NHS Foundation Trust
**Primary Subject Heading**:	Mental health
Secondary Subject Heading:	Research methods
Keywords:	Schizophrenia < psychotic disorders < PSYCHIATRY, PSYCHIATRY, Anomalous experiences, Functional outcome, Subjective outcome, Cognition

SCHOLARONE™
Manuscripts

Developing a dynamic model of anomalous experiences and function in young people with or without psychosis: a cross-sectional and longitudinal study protocol.

Abigail Wright* [a], Professor David Fowler [a b], Dr Kathryn Greenwood [a b]*.

[a] *University of Sussex, School of Psychology*

[b] *Sussex Partnership NHS Foundation Trust*

**correspondence to Abigail Wright: a.c.wright@sussex.ac.uk*

For peer review only

ABSTRACT

Introduction: Anomalous experiences are common within the general population, but the frequency and intensity is increased in young people with psychosis. Studies have demonstrated that perceptual biases towards noticing these phenomena plays a role, but the way one thinks about one's experience (metacognition) may also be relevant. Whilst poor metacognitive function has been theoretically associated with anomalous experiences, this relationship is currently unclear. However, metacognition may work along a continuum with various metacognitive levels, many of which have been demonstrated as impaired in psychosis. These metacognitive components may interact via processes that maintain poor metacognition across levels, and that potentially impact both what people do in their everyday lives (functional outcome) and how people feel about their everyday lives (subjective recovery outcome) in young people with psychosis compared to healthy control participants.

Methods and analysis: This study will investigate the association and contribution of metacognition to anomalous experiences and outcome measures cross-sectionally, and longitudinally in a 36-month follow-up. Firstly, young people with psychosis will be compared with healthy control participants on selected measures of anomalous experience, metacognition, and function, using ANCOVA to identify group differences. Next, the relationship between metacognitive components and processes will be explored, including processes connecting the different components, using regression analyses. Finally, mediation analyses will be used to assess the predictive value of metacognitive measures on outcome measures, both cross-sectionally and at longitudinally 36-months, whilst controlling for symptoms and cognition.

Ethics and dissemination: Ethical and Health Research Authority approval has been obtained through Camberwell St. Giles Research Ethics Committee (reference number: 17/LO/0055). This research project will be reported within a PhD thesis and submitted for journal publication. Once key predictive components of poor outcome in psychosis are identified, this study will develop a series of dynamic models to understand influences on outcome for young people with psychosis.

ARTICLE SUMMARY:

Strengths and limitations of this study:

- This is one of the first studies to assess metacognitive processes as predictors of functional outcome in young people with psychosis, and one of the first to assess metacognition as a longitudinal predictor of functional outcome in young people with psychosis sample.
- This study has used up-to-date paradigm within the field of metacognition to avoid biases.
- Due to length of follow-up period, it may be difficult to engage follow-up participants within this study. If re-contacting is difficult, the study may only have power to detect effects of the separate paths of the longitudinal analyses, not the full model.

Word count: 4688

INTRODUCTION

Anomalous experiences refer to a rich number of various psychic phenomena. These experiences can be divided into three main categories: anomalous self-experiences, the sense that you are not "real" (distortions in experience of self and being); anomalous perceptual experiences, hearing sounds which cannot be accounted for by the environment (distortions of sensory events in various domains; auditory, visual; touch; taste); and anomalous delusional beliefs, experiencing unusual thoughts or beliefs. These experiences may be common within the general population[1,2] but the frequency and intensity of these anomalous experiences is increased in those with psychosis or those with emerging severe mental health difficulties[3,4] and may predict psychotic symptoms at a later stage[5,6]. Research has suggested that anomalous self-experiences are suggested to precede and generate "surface-level" anomalous perceptual experiences (hallucinations)[7] and anomalous delusional beliefs may be

For peer review only - http://bmjopen.bmj.com/site/about/guidelines.xhtml

developed from anomalous experiences[8,9,10], suggesting a hierarchical framework between the anomalous delusional beliefs, experiences and self-experiences.

Many theories have been proposed to understand anomalous experiences, including source-monitoring deficits[11] and aberrant salience hypothesis[12]. Signal detection theory (SDT) has been the foundation to this research and studies using SDT have demonstrated that anomalous perceptual experiences are associated with perceptual signal detection biases[13, 14]; bias towards stating that a stimulus was present when it was in-fact absent. Such signal detection biases have been consistently shown within psychosis/psychosis-proneness literature[15, 16, 17] and suggest that top-down processes on (false) perception can lead to hallucinations or delusions[8, 18, 19].

Recent evidence suggests these signal detection biases are associated with metacognition in healthy students[20], and metacognition may therefore play a role in anomalous experiences. Metacognition is defined as "thinking about thinking"[21, 22], an abstract view of the object-level[23]. Literature has demonstrated that hallucinatory experiences and delusional beliefs/ideation have been associated with metacognition (overconfidence, specifically) in both clinical and non-clinical groups[24, 25, 26]. In particular, those with psychosis demonstrate more incorrect self-monitoring responses with higher confidence[27, 28], also present in those with a history of hallucinations[29], and those at high risk groups[30].

This research has not been consistent (see Gawęda et al., 2013[28]) as some studies did not control for objective performance, crucial for metacognitive efficiency scores[31, 32.] A recent controlled study demonstrated that individuals with FEP and those at-risk were more likely to misattribute an imagined action for a performed action, compared to healthy controls[30], but found no difference in misattribution of verbal or non-verbal actions this suggests the deficit in metacognition may be across several modalities. However, metacognitive ability (measured using meta-*d'*) is known to be modality-specific[33, 34] and anomalous perceptual-experiences (e.g. hallucinations) can vary in modalities[35]. It has also been acknowledged that auditory anomalous experiences are most common in psychosis[36, 37], all of which may suggest a modality-specific association with auditory or visual anomalous experiences and perception/metacognition. This present study will assess the modality-specific association between perceptual bias (signal detection bias) and metacognitions with anomalous perceptual experiences in visual and auditory modalities, whilst controlling for objective performance (see figure 1).

For peer review only - http://bmjopen.bmj.com/site/about/guidelines.xhtml

Limited research has assessed the association between anomalous self-experiences and metacognition, but it may be suggested metacognitive efficiency may also be associated with anomalous self-experiences, previously alluded to by Dokic and Martin[38]. This study will empirically test the association between anomalous self-experiences and perceptual biases and metacognition. Anomalous self-experiences and anomalous delusional beliefs have not been considered to be modality-specific, therefore these measures are hypothesised to be related to both visual and auditory perceptual signal detection biases and metacognitive ability.

[INSERT FIGURE 1 HERE]

Metacognition has been considered fractionated and can appear in many different forms, associated within a dynamic model[23, 39]. Three levels of metacognition have been proposed: i) Metacognitive efficiency: "knowing that you know"[40], and this level in particular may involve unconscious knowledge to generate a "feeling of knowing"[41] and has been shown to be modality-specific[33], which can be assessed by within-task confidence ratings, ii) Metacognitive experience: appraisal of one's experience or performance after an activity, and iii) Metacognitive knowledge: trait-like, stable beliefs about one's experience or abilities, on which Lysaker and colleagues have grounded their work (see Lysaker et al. 2014). These metacognitive levels may influence each other via metacognitive processes. Nelson and Narens[23] highlighted two processes: i) Metacognitive controlling processes, (i.e. such that knowledge is used to control, guide and correct ongoing action)[42], and ii) Metacognitive monitoring processes (i.e. monitoring of ongoing experience in order to recognise anomalies and update higher level beliefs)[43], which are important for accurate metacognitive functioning.

As metacognition works in a hierarchical fashion, it may be expected the poor metacognitive efficiency in psychosis, demonstrated above, can impact the next component on the continuum: metacognitive experience, via metacognitive processes. However, Gilleen, Greenwood and David[44] demonstrated that metacognitive experience is on average intact within a group of individuals with schizophrenia, whilst metacognitive knowledge, trait-like, stable beliefs about experience or abilities, is deficit. The dissociation between the different levels of metacognition may be the result of impaired metacognitive processes (self-reflection and set-shifting)[45], akin to various other studies[46, 47, 48]. Due to limited studies, it is currently unclear which metacognitive processes may be driving these metacognitive difficulties. This

For peer review only - http://bmjopen.bmj.com/site/about/guidelines.xhtml

study aims to further understand the difficulties in metacognitive processes in young people with psychosis and matched healthy controls within two models (figure 2 and 3).

[INSERT FIGURE 2 HERE]

[INSERT FIGURE 3 HERE]

From this, we will assess which metacognitive components may impact on functional outcome: a measurable aspect of an individual's specific activities of daily living. Research suggests functional outcome is predicted by neurocognition[49, 50], functional capacity (measured using real-life performance skills task)[51, 52, 53], negative symptoms[54], which has been demonstrated to show a synergistic interaction with cognition to impact functioning[55], and, importantly here, metacognition. Metacognitive ability, measured using the Metacognition Assessment Interview (MAI) or MAS[22, 56], appears to play a crucial role on functional outcome, independent of cognition and symptoms[57, 58, 59]. In a recent study, Davies, Fowler & Greenwood[60] demonstrated that metacognition partially mediates the relationship between cognition and functional capacity, and fully mediates the relationship between functional capacity and functional outcome. Limited research has assessed whether metacognitive processes are also relevant in maintaining poor functional outcome over time, independent of cognition. With this in mind, a metacognitive factor will be derived from all metacognitive variables above to explore the impact on functional outcome and capacity (see figure 4) and a longitudinal model will also explore the relationships over time.

[INSERT FIGURE 4 HERE]

Functional outcome has also been associated with subjective recovery outcome (self-rated outcome reflecting sense of wellbeing and quality of life)[61, 62]. Metacognitive capabilities were related to components of recovery beyond the effects of psychiatric symptoms, including aspects of quality of life[63, 64, 65]. There is a complex relationship between metacognition and functional and subjective outcome, this study these relationships to enable in-depth understanding of functional recovery.

This study aims to develop and test a series of dynamic models to understand (i) the nature of metacognitive deficits compared to healthy controls (ii) the relationship between metacognitive components (iii) and the influences of metacognition on anomalous experiences and objective/subjective functional outcome for young people with psychosis. If

For peer review only - http://bmjopen.bmj.com/site/about/guidelines.xhtml

these proposed models can be demonstrated empirically, this can help to understand and remediate poor outcome within psychosis.

Hypotheses

Hypothesis 1: Anomalous experiences will be associated with increased signal detection biases and poor metacognitive efficiency, and this relationship will be domain-specific.

Hypothesis 2: Metacognitive control processes will significantly predict metacognitive knowledge and metacognitive monitoring processes will significantly predict metacognitive experience. Metacognitive control processes will significantly predict metacognitive experience and metacognitive monitoring processes will significantly predict metacognitive efficiency.

Hypothesis 3: Metacognitive variables (metacognitive knowledge, metacognitive processes and metacognitive experience) will significantly predict outcome measures (functional capacity, functional outcome and subjective recovery outcome) in young people with and without psychosis, even after controlling for anomalous experiences, symptoms and IQ.

Hypothesis 4: Metacognitive variables (metacognitive knowledge, metacognitive knowledge processes and metacognitive experience) will significantly predict outcome measures (functional capacity and functional outcome) at 36-month longitudinal follow-up of participants in young people with psychosis, even after controlling for symptoms and IQ.

METHODS

Design: This is a cross-sectional and longitudinal study. This cross-sectional aspect will explore (i) deficits in metacognition between young people with psychosis and healthy controls; (ii) interrelations between different metacognitive components and processes across the whole sample and (iii) the contribution of specific novel metacognitive variables to anomalous experiences and outcomes, and (iv) a longitudinal aspect will identify whether metacognition predicts experiences and outcomes at 36-month follow-up period (psychosis sample only).

Participants:

Seventy-three young people with psychosis will be recruited. This sample will be made up of a convenience sample from first episode services and the remaining individuals will be those

For peer review only - http://bmjopen.bmj.com/site/about/guidelines.xhtml

re-recruited from a previous first episode psychosis (FEP) sample (previous N=80), to take part in the main cross-sectional study and longitudinal follow-up aspect. Participants from the previous FEP study have baseline data on metacognition, functional capacity, functional outcome, symptoms and cognition[60]. This data will form the baseline data for the longitudinal analysis. All participants with psychosis will be 18 – 40 years of age, able to read and communicate in English and receiving treatment for psychosis with a UK Early Intervention in Psychosis services at first assessment. Participants with organic causes for psychosis or those with a diagnosis of substance use disorder will be excluded.

Seventy-three healthy control participants will be recruited as a comparison group. These participants will be 18-40 years of age, able to read and communicate in English and matched on age, gender and education with the psychosis sample. Participants will be recruited through advertisement within the local community, e.g. in libraries, cafes and on social media. Participants with current mental health problems or history of psychosis will be excluded following screening questions: i) Are you currently experiencing any mental health difficulties? ii) Are you on any psychotropic medication/substances? iii) Have you been in contact with psychological or psychiatric services for psychological problems? iv) Has anyone in your immediate family experienced an episode of psychosis? E.g. parents, siblings. If healthy control answered yes to any of the questions, these participants were deemed ineligible to take part in the study.

Any participant with hearing or sight problems which cannot be will be excluded. Data collection will be undertaken within an NHS building or community setting between 10th March 2017 and 4th May 2018. The end date of the study is 18th September 2018.

Patient and Public Involvement

Patient and Public Involvement is primarily via the Psychosis Interest Group run by Service User and Carer Involvement Coordinator at the Sussex Partnership NHS Foundation Trust Research and Development department, and service users within the Psychosis Theme Group (PTG). The first author met with the PTG to consult on the development of this project, including the design, methods, and procedure of the project. The lived experience group have viewed all the measures, including the two main computer tasks (visual and auditory tasks), and provided extensive feedback which has been incorporated into this project. Study participants who consent to receiving the study results will receive these by post/email. The

For peer review only - http://bmjopen.bmj.com/site/about/guidelines.xhtml

first author and the Psychosis Theme Group will continue to meet to consult on recruitment procedures, and on the plans for dissemination to service user groups following data analysis.

Cross-sectional measures:

Anomalous experience measures

Anomalous perceptual experiences: Multimodal Unusual Sensory Experiences Questionnaire[35]. This measures anomalous perceptual/sensory experiences with 6 subscales. Both the full scale and subscales have been demonstrated as possessing good reliability (auditory r=0.72, visual r=0.72), good validity between clinical and non-clinical group (Cohen's d = 0.96) and significance with all other anomalous experience scale, and internal consistency (auditory α=0.82, visual α=0.88). The auditory and visual subscales will be used for this study which each provide a combined score for presence and frequency of anomalous experiences.

Anomalous self-experiences: Cambridge depersonalisation scale (trait and state versions)[66]. The trait version includes 29 items measuring frequency (Never to All the time) and duration (Few seconds to More than a week) of anomalous self-experiences over the last 6 months. It demonstrates high internal consistency (>0.6), good validity with other scales (r=0.8) and good reliability (α= 0.89)[66], and is useful for assessing depersonalisation in a schizophrenia group[67]. The state version includes 22 items measuring anomalous self-experiences within-the-moment on a scale of intensity from 0-100. Subscales of the CDS are 'alienation from surroundings', 'anomalous subjective recall', 'emotional numbing' and 'anomalous body experience'.

Anomalous delusional beliefs: Schizotypal Symptom Inventory[68]. This measure assesses residual psychotic symptoms, providing a total score with separate subscales for paranoia, anomalous experience and social anxiety[69]. This present study will use the paranoia subscale as a measure of anomalous delusional beliefs and the anomalous experience subscale as a measure of anomalous perceptual experiences, to confirm MUSEQ data assessing anomalous perceptual experiences. This scale demonstrates high internal consistency (non-clinical sample α=0.87 and clinical sample α=0.92), good validity with other scales (PANSS with total r = 0.59 and paranoia r=0.6) and good test-retest reliability (0.85 for total and 0.6-0.84 for subscales).

For peer review only - http://bmjopen.bmj.com/site/about/guidelines.xhtml

Metacognition

Metacognitive efficiency

This encompasses separate computer –based visual and auditory detection task. The critical task in both paradigms is to make two forced-choice binary judgments of whether a visual or auditory stimulus (dot or tone) was present or absent (first judgement) within a noisy picture or presentation of white noise, and whether this was associated with high confidence or low confidence in the first judgement (second judgement).

The first judgement can be used to calculate a score for signal detection perceptual sensitivity and perceptual signal detection bias. *Perceptual sensitivity* (*d'*): the ability to correct report whether the stimulus (dot/tone) was either present or absent. Signal detection theory (SDT) posits that detection making involves depicting whether certain waveforms called signals may or may not be embedded within background 'noise', using internal responses[70]. *Perceptual bias* (*B*) is the tendency to report one decision over the other, e.g. stating the stimuli was present when it was in-fact absent. The internal responses from perceptual sensitivity score is then compared to a decision criterion (c) so all evidence above criteria elicits a response of 'present' compared to below the criteria elicits the response of 'absent'[71]. The perceptual sensitivity score and the decision criterion can be fitted to an empirical Receiver Operating Characteristic (ROC) curve, enabling us to assess perceptual biases in responses, e.g. whether someone was more or less likely to report stimuli as being present based on a lower/higher decision criterion.

The second judgement can be used to calculate a score for metacognitive sensitivity and metacognitive efficiency. *Metacognitive sensitivity* (meta-*d'*) is the ability to discriminate between correct and incorrect judgments. Alike to the SDT above, metacognitive sensitivity is based on assessment of sensitivity (how well confidence ratings discriminate correct from incorrect trials) and response bias (overall propensity for reporting high confidence). However, this score from the second judgement must take into account first judgment performance[72]. Meta-*d'* indicates the *d'* that would have been predicted to result in the confidence rating assuming the signal detection theory. Optimal metacognitive sensitivity is when perceptual sensitivity score is matched. Meta-*d'* greater or less than *d'* indicates metacognition is better or worse than *d'* [34]. *Metacognitive efficiency* is one's ability to discriminate between one's own correct or incorrect perceptual decision, whilst taking into account objective performance[32]. This is calculated as meta-*d'*/*d'*. Metacognitive efficiency

For peer review only - http://bmjopen.bmj.com/site/about/guidelines.xhtml

(meta-*d'*/*d'*) was chosen as a more robust form of perceptual metacognition, over metacognitive sensitivity (meta-*d'*) which was previously used by researchers[73].

Performance on the first judgment will be held constant throughout the task and across participants (using a 1-up-2-down staircase procedure) to ensure that metacognitive ability is measured independent of task performance and produces valid scores.

Studies including signal detection tasks typically involve a large number of trials (~400), to avoid statistical bias and large variance in scores[74]. However, following a pilot study, we reduced the trials to 200, whilst maintaining reliable data. To ensure the feasibility of conducting this study within a clinical population, who may present difficulty with attention and concentration[75], the two computer tasks have been developed from a pilot study within a healthy student population[76].

Alongside this, participants will be asked to prospectively and retrospectively rate their performance on the detection tasks. These ratings will be used to assess metacognitive knowledge and metacognitive experience.

Metacognitive knowledge: The prospective rating ("How well do you think you will perform overall? For instance, if you think you will get it right every time, select 100 (all correct). If you think you will be correct none of the time, select 0 (none correct). You can select any value in between zero and 100 to indicate what percentage you think you will correctly identify") will assess task-related metacognitive knowledge.

Metacognitive experience: The retrospective rating ("How well do you think you performed overall on the task? For instance, if you think you were right every time, select 100 (all correct). If you think you were correct none of the time, select 0 (none correct). You can select any value in between zero and 100 to indicate what percentage you think you correctly identified") will assess task-related metacognitive experience.

Metacognitive ability: This will be assessed using the Metacognitive Assessment Interview[56], to measure metacognitive knowledge. This measure assesses the ability to understand "the self" and "the other"; termed as one multidimensional construct as 'metacognition'. This measure has demonstrated good inter-rater reliability and internal consistency (α=0.90 for total metacognition), factorial validity, and reliability (r=0.62 to 0.90)[56].

Metacognitive processes (monitoring and control): Monitoring processes will be assessed using self-reflectivity subscale of Cognitive insight scale[77]. This measure possesses good

For peer review only - http://bmjopen.bmj.com/site/about/guidelines.xhtml

internal consistency (α=0.68), convergent validity (with SUMD-A delusions, r= -0.67)[77] and test-retest reliability (r=0.90)[78]. Studies have demonstrated this measure is appropriate to assess metacognitive monitoring[45, 79]. Control processes will be assessed via set-shifting using trail-making task[80] part B-A. This measure possesses good internal consistency (TMT-A α=0.39, TMT-B α=0.71), convergent validity (with Task Switching Paradigm r=0.32)[81], and good reliability of other forms of TMT (r=0.78)[82]. This measure is appropriate to assess metacognitive control processes[45].

Functional outcome

Functional outcome: Time Use Survey (adapted from Short[83]) provides a retrospectively rated objective measure for hours spent engaging in structured activity per week[84]. This measure has been used within a First Episode Psychosis sample to assess functioning[85, 86]. This measure has good reliability (inter-rater reliability at 0.99)[85], coder reliability (89% accuracy)[87], good validity as differences in Time-Use have been demonstrated between different stages of psychosis, representing social recovery in psychosis[85], validity as TUS is comparable to studies using functioning measures[69].

Functional capacity: The UCSD Performance-Based Skills Assessment[53] provides a total score for real-life performance skills based on simulated tasks. It was adapted to be applicable for UK participants. This measure demonstrates high internal consistency (α=0.88), good validity with other scales (DAFS r=0.86) and good test-retest reliability (r=0.91)[88].

Subjective recovery outcome: The Questionnaire of Process of Recovery[89] provides a score for an individual's subjective functioning (psychosis participants only). This scale has two subscales: intrapersonal items related to hope, empowerment, confidence, and interpersonal related to connectedness with others, others help/care, reliance. This possesses good internal consistency (intrapersonal subscale, α=.94, interpersonal subscale, α=.77), construct validity (GHQ total score: intra, r=-.83 inter, r=.52) and reliability (intra, r=.87, inter, r.77)[89].

Covariates

Symptom severity: This assesses symptoms of psychosis using Positive and Negative Syndrome Scale[90] (clinical participants only), which is the mostly widely used standardised instrument for assessing symptom severity in schizophrenia[91]. This measure has

For peer review only - http://bmjopen.bmj.com/site/about/guidelines.xhtml

demonstrated good reliability and validity, and appropriate inverse correlations between positive and negative subscales[92]. This measure has good internal consistency (agreement for PANSS items r=0.69 – 0.94), construct validity (between PANSS and Andreasen rating system, r=0.77) and reliability (interrater correlations for PANSS scales ranged from 0.83 to 0.87)[92].

Cognitive ability: This includes verbal IQ: Vocabulary task[93] is a measure of an individual's verbal knowledge and fund of information. This measure as good internal consistency (correlation with other cognitive measures range r=0.54 to 0.79), construct validity (with WASI-III, r=0.88) and reliability (0.90 to 0.89) and test-retest (0.88)[93]; and performance IQ: Matrix reasoning task[93] is a measure of individual's ability to mentally manipulate abstract symbols and perceive the relationship among them. This measure as good internal consistency (correlation with other cognitive measures range r=0.59 to 0.63), construct validity (with WASI-III, r=0.66) and reliability (0.88 to 0.96) and test-retest (0.76)[93].

Longitudinal measures:

For participants who have baseline data, this comprises of following measures outlined above: metacognitive knowledge and metacognitive monitoring processes; functional outcome and functional capacity; and all covariates.

ANALYSIS

Sample size: Two sample size estimates have been combined to ensure the analyses have sufficient power to detect effects.

Firstly, sample size to detect differences between individuals with psychosis and healthy control participants on metacognitive efficiency. Using G power with .8 power, .57 effect size based on a previous metacognitive efficiency task in psychosis[94] and 0.05 alpha, the proposed total sample size is 28 (14 per group). Secondly, a regression analysis to assess the predictive value of metacognitive knowledge, processes and experience on outcome measures (controlling for symptoms and IQ) will be conducted. G power estimation was used for a power calculation based on a power of .80, effect size of .313[60] and alpha of .05. This suggested for 6 predictors a total of 55 participants are required.

In terms of the mediation analysis, power estimation was calculated using the monte carlo method to estimate power for complex mediation models (see Thoemmes, MacKinnon & Resier[95]). Using fixed parameters from Davies, Fowler and Greenwood[60] and power at .8, this

For peer review only - http://bmjopen.bmj.com/site/about/guidelines.xhtml

suggested a total sample size of 146 participants to detect mediation effects, outlined in the above model.

In terms of the longitudinal analysis, as many of the original sample (n=80) as possible will be followed up to maximise statistical power.

Planned data analysis: Data will be double entered and checked for accuracy, and checked for outliers.

Missing data will be considered as 'Missing at random' (MAR), which means the missing variables are related to additional observed variables within the data, but values of missing data itself. Missing data will be treated according to best practice[96]. Principled missing data methods will be used which combine available information from the observed data to estimate the population parameters and/or the missing data mechanism[97]. Full information maximum likelihood (FIML) involves using all the observed data and creating values for missing data using maximum likelihood estimations. This works well provided that the model for the complete data is realistic[98]. FIML will be used within this study which is considered most appropriate for MAR data and for mediation analyses[99]. Quantitative data (including demographic information) will be reported using descriptive statistics, e.g. means and standard deviations.

Data will be analysed using SPSS and Mplus software. Group differences of all metacognitive measures and perceptual signal detection biases between young people with psychosis and healthy controls will be assessed, controlling for age and IQ. Linear regression analyses will be used to assess how metacognition (and perceptual signal detection biases) predicts anomalous experiences. Cross-sectional and longitudinal predictive analyses will be conducted, including regression analyses which will assess whether metacognitive components; knowledge, experience, efficiency are predicted by metacognitive processes. A mediation analysis will be used to whether functional capacity, functional outcome and subjective recovery outcome are predicted by metacognition and metacognitive processes, whilst controlling for anomalous experiences, symptoms and cognition. These analyses will include bootstrapped bias-corrected confidence intervals.

DISCUSSION

Our assumption is that metacognitive variables predict, and maintain, anomalous events, poor objective and subjective recovery outcome in young people with and without psychosis.

For peer review only - http://bmjopen.bmj.com/site/about/guidelines.xhtml

Particularly in young people with psychosis, metacognitive deficits may predict long-term functional outcome.

The study results will be an important addition to the literature and for clinicians for four main reasons: i) this study tests a proposed model from previous literature which may help understand poor functional outcome in psychosis, ii) from this, novel intervention studies can be developed to tackle the potential metacognitive deficits in psychosis which predict this poor functional outcome, iii) this is one of the first studies to assess metacognition as a longitudinal predictor of functional outcome in young people with psychosis sample, and finally iv) our studies use up-to-date paradigms within the field of metacognition to avoid biases.

Limitations

A foreseeable limitation is that the First Episode Psychosis sample will be comprised of both a previous FEP sample and new participants who are currently engaged in EIS in Sussex. Therefore, individuals will be at various stages of their recovery and support from the Early Intervention Services which adds variation in terms of symptoms and recovery. With this in mind, symptoms will be controlled in the main analysis. However, this will enable exploration of factors which predict this variation. Due to length of follow-up period, another limitation may be the difficulty in re-recruiting these participants into the study. If re-contacting is difficult, the model may not have full power. If so, the individual paths of the model in the longitudinal analyses will be explored.

ETHICS AND DISSEMINATION

This study has been reviewed and approved by Camberwell St. Giles Research Ethics Committee (reference number: 17/LO/0055). The data will be stored securely in accordance with usual NHS procedures and data will be governed by the sponsor: University of Sussex. This research project will be reported within a PhD thesis, and will be written up for publication in scientific journals.

REFERENCES

1. Bell V, Halligan PW, Ellis HD. The Cardiff Anomalous Perceptions Scale (CAPS): a new validated measure of anomalous perceptual experience. Schizophrenia bulletin. 2005 Oct 29;32(2):366-77.

For peer review only - http://bmjopen.bmj.com/site/about/guidelines.xhtml

2. Kelleher I, Connor D, Clarke MC, Devlin N, Harley M, Cannon M. Prevalence of psychotic symptoms in childhood and adolescence: a systematic review and meta-analysis of population-based studies. Psychological medicine. 2012 Sep;42(9):1857-63.
3. Brett CM, Johns LC, Peters EP, McGuire PK. The role of metacognitive beliefs in determining the impact of anomalous experiences: a comparison of help-seeking and non-help-seeking groups of people experiencing psychotic-like anomalies. Psychological Medicine. 2009 Jun;39(6):939-50.
4. Reininghaus U, Kempton MJ, Valmaggia L, Craig TK, Garety P, Onyejiaka A, Gayer-Anderson C, So SH, Hubbard K, Beards S, Dazzan P. Stress sensitivity, aberrant salience, and threat anticipation in early psychosis: an experience sampling study. Schizophrenia bulletin. 2016 Feb 1;42(3):712-22.
5. Yung AR, Phillips LJ, Yuen HP, McGorry PD. Risk factors for psychosis in an ultra high-risk group: psychopathology and clinical features. Schizophrenia research. 2004 Apr 1;67(2-3):131-42.
6. Yung AR, Phillips LJ, Yuen HP, Francey SM, McFarlane CA, Hallgren M, McGorry PD. Psychosis prediction: 12-month follow up of a high-risk ("prodromal") group. Schizophrenia research. 2003 Mar 1;60(1):21-32.
7. Nelson B, Parnas J, Sass LA. Disturbance of minimal self (ipseity) in schizophrenia: clarification and current status.
8. Fletcher PC, Frith CD. Perceiving is believing: a Bayesian approach to explaining the positive symptoms of schizophrenia. Nature Reviews Neuroscience. 2009 Jan;10(1):48.
9. Garety PA, Kuipers E, Fowler D, Freeman D, Bebbington PE. A cognitive model of the positive symptoms of psychosis. Psychological medicine. 2001 Feb;31(2):189-95.
10. Freeman D, Garety PA, Kuipers E, Fowler D, Bebbington PE. A cognitive model of persecutory delusions. British Journal of Clinical Psychology. 2002 Nov;41(4):331-47.
11. Nelson B, Whitford TJ, Lavoie S, Sass LA. What are the neurocognitive correlates of basic self-disturbance in schizophrenia?: Integrating phenomenology and neurocognition. Part 1 (Source monitoring deficits). Schizophrenia research. 2014 Jan 1;152(1):12-9.
12. Nelson B, Whitford TJ, Lavoie S, Sass LA. What are the neurocognitive correlates of basic self-disturbance in schizophrenia?: integrating phenomenology and

For peer review only - http://bmjopen.bmj.com/site/about/guidelines.xhtml

neurocognition: part 2 (aberrant salience). Schizophrenia research. 2014 Jan 1;152(1):20-7.

13. Bentall RP, Slade PD. Reality testing and auditory hallucinations: a signal detection analysis. British Journal of Clinical Psychology. 1985 Sep;24(3):159-69.
14. Pajani A, Kok P, Kouider S, de Lange FP. Spontaneous activity patterns in primary visual cortex predispose to visual hallucinations. Journal of Neuroscience. 2015 Sep 16;35(37):12947-53.
15. Davies DJ, Teufel C, Fletcher PC. Anomalous perceptions and beliefs are associated with shifts toward different types of prior knowledge in perceptual inference. Schizophrenia bulletin. 2017 Dec 27.
16. Mussgay L, Hertwig R. Signal detection indices in schizophrenics on a visual, auditory, and bimodal continuous performance test. Schizophrenia Research. 1990 Oct 1;3(5-6):303-10.
17. Teufel C, Subramaniam N, Dobler V, Perez J, Finnemann J, Mehta PR, Goodyer IM, Fletcher PC. Shift toward prior knowledge confers a perceptual advantage in early psychosis and psychosis-prone healthy individuals. Proceedings of the National Academy of Sciences. 2015 Oct 27;112(43):13401-6.
18. Cook JL, Barbalat G, Blakemore SJ. Top-down modulation of the perception of other people in schizophrenia and autism. Frontiers in human neuroscience. 2012 Jun 15;6:175.
19. Powers AR, Mathys C, Corlett PR. Pavlovian conditioning–induced hallucinations result from overweighting of perceptual priors. Science. 2017 Aug 11;357(6351):596-600.
20. Sherman MT, Seth AK, Barrett AB, Kanai R. Prior expectations facilitate metacognition for perceptual decision. Consciousness and cognition. 2015 Sep 1;35:53-65.
21. Flavell JH. Metacognition and cognitive monitoring: A new area of cognitive–developmental inquiry. American psychologist. 1979 Oct;34(10):906.
22. Semerari A, Carcione A, Dimaggio G, Falcone M, Nicolò G, Procacci M, Alleva G. How to evaluate metacognitive functioning in psychotherapy? The metacognition assessment scale and its applications. Clinical Psychology & Psychotherapy. 2003 Jul;10(4):238-61.
23. Nelson TO. Metamemory: A theoretical framework and new findings. InPsychology of learning and motivation 1990 Jan 1 (Vol. 26, pp. 125-173). Academic Press.

For peer review only - http://bmjopen.bmj.com/site/about/guidelines.xhtml

24. Eisenacher S, Rausch F, Ainser F, Mier D, Veckenstedt R, Schirmbeck F, Lewien A, Englisch S, Andreou C, Moritz S, Meyer-Lindenberg A. Investigation of metamemory functioning in the at-risk mental state for psychosis. Psychological medicine. 2015 Nov;45(15):3329-40.
25. Moritz S, Woodward TS, Whitman JC, Cuttler C. Confidence in errors as a possible basis for delusions in schizophrenia. The Journal of nervous and mental disease. 2005 Jan 1;193(1):9-16.
26. Warman DM. Reasoning and delusion proneness: confidence in decisions. The Journal of nervous and mental disease. 2008 Jan 1;196(1):9-15.
27. Gawęda Ł, Moritz S, Kokoszka A. Impaired discrimination between imagined and performed actions in schizophrenia. Psychiatry Research. 2012 Jan 30;195(1-2):1-8.
28. Gawęda Ł, Woodward TS, Moritz S, Kokoszka A. Impaired action self-monitoring in schizophrenia patients with auditory hallucinations. Schizophrenia research. 2013 Mar 1;144(1-3):72-9.
29. Gaweda L, Mikuła J, Szelenbaum W, Kokoszka A. Towards a cognitive model of hallucinations in the course of alcohol dependence? A source monitoring-based pilot study. Psychological medicine. 2014 Oct;44(13):2763-73.
30. Gaweda L, Li E, Lavoie S, Whitford TJ, Moritz S, Nelson B. Impaired action self-monitoring and cognitive confidence among ultra-high risk for psychosis and first-episode psychosis patients. European Psychiatry. 2018 Jan 1;47:67-75.
31. Balzan RP. Overconfidence in psychosis: The foundation of delusional conviction?. Cogent Psychology. 2016 Dec 31;3(1):1135855.
32. Fleming SM, Lau HC. How to measure metacognition. Frontiers in human neuroscience. 2014 Jul 15;8:443.
33. Fleming SM, Ryu J, Golfinos JG, Blackmon KE. Domain-specific impairment in metacognitive accuracy following anterior prefrontal lesions. Brain. 2014 Aug 6;137(10):2811-22.
34. Morales J, Lau H, Fleming SM. Domain-specific patterns of activity support metacognition in human prefrontal cortex. bioRxiv. 2017 Jan 1:172445.
35. Mitchell CA, Maybery MT, Russell-Smith SN, Collerton D, Gignac GE, Waters F. The Structure and Measurement of Unusual Sensory Experiences in Different Modalities: The Multi-Modality Unusual Sensory Experiences Questionnaire (MUSEQ). Frontiers in psychology. 2017 Aug 11;8:1363.
36. Shergill SS, Murray RM, McGuire PK. Auditory hallucinations: a review of

For peer review only - http://bmjopen.bmj.com/site/about/guidelines.xhtml

psychological treatments. Schizophrenia research. 1998 Aug 17;32(3):137-50.

37. Waters F, Allen P, Aleman A, Fernyhough C, Woodward TS, Badcock JC, Barkus E, Johns L, Varese F, Menon M, Vercammen A. Auditory hallucinations in schizophrenia and nonschizophrenia populations: a review and integrated model of cognitive mechanisms. Schizophrenia bulletin. 2012 Mar 23;38(4):683-93.
38. Dokic J, Martin JR. Felt reality and the opacity of perception. Topoi. 2017 Jun 1;36(2):299-309.
39. Shea N, Boldt A, Bang D, Yeung N, Heyes C, Frith CD. Supra-personal cognitive control and metacognition. Trends in cognitive sciences. 2014 Apr 1;18(4):186-93.
40. Sherman MT, Barrett AB, Kanai R. Inferences about consciousness using subjective reports of confidence. Behavioral methods in consciousness research. 2015:87-106.
41. Nelson TO. A comparison of current measures of the accuracy of feeling-of-knowing predictions. Psychological bulletin. 1984 Jan;95(1):109.
42. Wells A, Purdon C. Metacognition and cognitive-behaviour therapy: a special issue. Clinical Psychology & Psychotherapy: An International Journal of Theory & Practice. 1999 May;6(2):71-2.
43. Efklides A. Metacognition: Defining its facets and levels of functioning in relation to self-regulation and co-regulation. European Psychologist. 2008 Jan;13(4):277-87.
44. Gilleen J, Greenwood K, David AS. The role of memory in awareness of memory deficits in Alzheimer's disease, schizophrenia, and brain injury. Journal of clinical and experimental neuropsychology. 2014 Jan 2;36(1):43-57.
45. Gilleen J, David A, Greenwood K. Self-reflection and set-shifting mediate awareness in cognitively preserved schizophrenia patients. Cognitive neuropsychiatry. 2016 May 3;21(3):185-96.
46. Engh JA, Friis S, Birkenaes AB, Jónsdóttir H, Klungsøyr O, Ringen PA, Simonsen C, Vaskinn A, Opjordsmoen S, Andreassen OA. Delusions are associated with poor cognitive insight in schizophrenia. Schizophrenia bulletin. 2009 Jan 27;36(4):830-5.
47. Medalia A, Lim RW. Self-awareness of cognitive functioning in schizophrenia. Schizophrenia research. 2004 Dec 1;71(2-3):331-8.
48. Warman DM, Martin JM. Cognitive insight and delusion proneness: an investigation using the Beck Cognitive Insight Scale. Schizophrenia research. 2006 Jun 1;84(2-3):297-304.
49. Green MF. What are the functional consequences of neurocognitive deficits in schizophrenia?. The American journal of psychiatry. 1996 Mar 1;153(3):321.

For peer review only - http://bmjopen.bmj.com/site/about/guidelines.xhtml

50. Green MF, Kern RS, Braff DL, Mintz J. Neurocognitive deficits and functional outcome in schizophrenia: are we measuring the "right stuff"?. Schizophrenia bulletin. 2000 Jan 1;26(1):119-36.
51. Bowie CR, Reichenberg A, Patterson TL, Heaton RK, Harvey PD. Determinants of real-world functional performance in schizophrenia subjects: correlations with cognition, functional capacity, and symptoms. American Journal of Psychiatry. 2006 Mar 1;163(3):418-25.
52. Bowie CR, Leung WW, Reichenberg A, McClure MM, Patterson TL, Heaton RK, Harvey PD. Predicting schizophrenia patients' real-world behavior with specific neuropsychological and functional capacity measures. Biological psychiatry. 2008 Mar 1;63(5):505-11.
53. Patterson TL, Goldman S, McKibbin CL, Hughs T, Jeste DV. UCSD Performance-Based Skills Assessment: development of a new measure of everyday functioning for severely mentally ill adults. Schizophrenia bulletin. 2001 Jan 1;27(2):235-45.
54. Milev P, Ho BC, Arndt S, Andreasen NC. Predictive values of neurocognition and negative symptoms on functional outcome in schizophrenia: a longitudinal first-episode study with 7-year follow-up. American Journal of Psychiatry. 2005 Mar 1;162(3):495-506.
55. Greenwood KE, Landau S, Wykes T. Negative symptoms and specific cognitive impairments as combined targets for improved functional outcome within cognitive remediation therapy. Schizophrenia Bulletin. 2005 Jan 1;31(4):910-21.
56. Semerari A, Cucchi M, Dimaggio G, Cavadini D, Carcione A, Battelli V, Nicolò G, Pedone R, Siccardi T, Stefania D, Ronchi P. The development of the Metacognition Assessment Interview: Instrument description, factor structure and reliability in a non-clinical sample. Psychiatry Research. 2012 Dec 30;200(2-3):890-5.
57. James AV, Hasson-Ohayon I, Vohs J, Minor KS, Leonhardt BL, Buck KD, George S, Lysaker PH. Metacognition moderates the relationship between dysfunctional self-appraisal and social functioning in prolonged schizophrenia independent of psychopathology. Comprehensive psychiatry. 2016 Aug 1;69:62-70.
58. Lysaker PH, McCormick BP, Snethen G, Buck KD, Hamm JA, Grant M, Nicolò G, Dimaggio G. Metacognition and social function in schizophrenia: associations of mastery with functional skills competence. Schizophrenia Research. 2011 Sep 1;131(1-3):214-8.

For peer review only - http://bmjopen.bmj.com/site/about/guidelines.xhtml

59. Lysaker PH, Leonhardt BL, Pijnenborg M, van Donkersgoed R, de Jong S, Dimaggio G. Metacognition in schizophrenia spectrum disorders: methods of assessment and associations with neurocognition, symptoms, cognitive style and function. Isr J Psychiatry Relat Sci. 2014 Jan 1;51(1):54-61.

60. Davies G, Fowler D, Greenwood K. Metacognition as a mediating variable between neurocognition and functional outcome in first episode psychosis. Schizophrenia bulletin. 2016 Sep 2;43(4):824-32.

61. Malla A, Payne J. First-episode psychosis: psychopathology, quality of life, and functional outcome. Schizophrenia bulletin. 2005 Jan 1;31(3):650-71.

62. Provencher HL, Gregg R, Mead S, Mueser KT. The role of work in the recovery of persons with psychiatric disabilities. Psychiatric rehabilitation journal. 2002;26(2):132.

63. Kukla M, Lysaker PH, Salyers MP. Do persons with schizophrenia who have better metacognitive capacity also have a stronger subjective experience of recovery?. Psychiatry Research. 2013 Oct 30;209(3):381-5.

64. Phalen PL, Viswanadhan K, Lysaker PH, Warman DM. The relationship between cognitive insight and quality of life in schizophrenia spectrum disorders: symptom severity as potential moderator. Psychiatry research. 2015 Dec 30;230(3):839-45.

65. Lysaker PH, Carcione A, Dimaggio G, Johannesen JK, Nicolò G, Procacci M, Semerari A. Metacognition amidst narratives of self and illness in schizophrenia: associations with neurocognition, symptoms, insight and quality of life. Acta psychiatrica scandinavica. 2005 Jul;112(1):64-71.

66. Sierra M, Berrios GE. The Cambridge Depersonalisation Scale: a new instrument for the measurement of depersonalisation. Psychiatry research. 2000 Mar 6;93(2):153-64.

67. Perona-Garcelán S, Carrascoso-López F, García-Montes JM, Vallina-Fernández O, Pérez-Álvarez M, Ductor-Recuerda MJ, Salas-Azcona R, Cuevas-Yust C, Gómez-Gómez MT. Depersonalization as a mediator in the relationship between self-focused attention and auditory hallucinations. Journal of Trauma & Dissociation. 2011 Oct 1;12(5):535-48.

68. Hodgekins J, Coker S, Freeman D, Ray-Glover K, Bebbington P, Garety P, Kuipers E, Dunn G, Fowler D. Assessing levels of subthreshold psychotic symptoms in the recovery phase: the Schizotypal Symptoms Inventory (SSI). Journal of Experimental Psychopathology. 2012 Oct;3(4):jep-021211.

69. Hodgekins J. *Investigating the psychological typology of social recovery in individuals with*

first episode psychosis (Doctoral dissertation, University of East Anglia).

70. Green DMSwets JA. Signal detection theory and psychophysics.
71. Maniscalco B, Peters MA, Lau H. Heuristic use of perceptual evidence leads to dissociation between performance and metacognitive sensitivity. Attention, Perception, & Psychophysics. 2016 Apr 1;78(3):923-37.
72. Maniscalco B, Lau H. Signal detection theory analysis of type 1 and type 2 data: meta-d′, response-specific meta-d′, and the unequal variance SDT model. InThe cognitive neuroscience of metacognition 2014 (pp. 25-66). Springer, Berlin, Heidelberg.
73. Maniscalco B, Lau H. A signal detection theoretic approach for estimating metacognitive sensitivity from confidence ratings. Consciousness and cognition. 2012 Mar 1;21(1):422-30.
74. Barrett AB, Dienes Z, Seth AK. Measures of metacognition on signal-detection theoretic models. Psychological methods. 2013 Dec;18(4):535.
75. Silverman J. The problem of attention in research and theory in schizophrenia. Psychological Review. 1964 Sep;71(5):352.
76. Wright AC, Fowler D, Greenwood K. Understanding the association between anomalous experiences and perceptual and metacognitive ability within the general population: a pilot study. (in preparation).
77. Beck AT, Baruch E, Balter JM, Steer RA, Warman DM. A new instrument for measuring insight: the Beck Cognitive Insight Scale. Schizophrenia research. 2004 Jun 1;68(2-3):319-29.
78. Pedrelli P, McQuaid JR, Granholm E, Patterson TL, McClure F, Beck AT, Jeste DV. Measuring cognitive insight in middle-aged and older patients with psychotic disorders. Schizophrenia research. 2004 Dec 1;71(2-3):297-305.
79. David AS, Bedford N, Wiffen B, Gilleen J. Failures of metacognition and lack of insight in neuropsychiatric disorders. InThe Cognitive Neuroscience of Metacognition 2014 (pp. 345-365). Springer, Berlin, Heidelberg.
80. Reitan RM. Trail making test results for normal and brain-damaged children. Perceptual and motor skills. 1971 Oct;33(2):575-81.
81. Sanchez-Cubillo I, Perianez JA, Adrover-Roig D, Rodriguez-Sanchez JM, Rios-Lago M, Tirapu JE, Barcelo F. Construct validity of the Trail Making Test: role of task-switching, working memory, inhibition/interference control, and visuomotor abilities. Journal of the International Neuropsychological Society. 2009 May;15(3):438-50.

For peer review only - http://bmjopen.bmj.com/site/about/guidelines.xhtml

82. Franzen MD, Paul D, Iverson GL. Reliability of alternate forms of the trail making test. The Clinical Neuropsychologist. 1996 Apr 1;10(2):125-9.
83. Short S. Review of the UK 2000 Time Use Survey. London: Office for National Statistics. 2006 Jul.
84. Fowler D, Hodgekins J, Howells L, Millward M, Ivins A, Taylor G, Hackmann C, Hill K, Bishop N, Macmillan I. Can targeted early intervention improve functional recovery in psychosis? A historical control evaluation of the effectiveness of different models of early intervention service provision in Norfolk 1998–2007. Early Intervention in Psychiatry. 2009 Nov;3(4):282-8.
85. Hodgekins J, Birchwood M, Christopher R, Marshall M, Coker S, Everard L, Lester H, Jones P, Amos T, Singh S, Sharma V. Investigating trajectories of social recovery in individuals with first-episode psychosis: a latent class growth analysis. The British Journal of Psychiatry. 2015 Dec;207(6):536-43.
86. Cella M, Edwards C, Wykes T. A question of time: A study of time use in people with schizophrenia. Schizophrenia research. 2016 Oct 1;176(2-3):480-4.
87. Short S. The United Kingdom 2000 Time Use Survey. Office for National Statistics, Newport, UK. 2003.
88. Harvey PD, Velligan DI, Bellack AS. Performance-based measures of functional skills: usefulness in clinical treatment studies. Schizophrenia bulletin. 2007 May 9;33(5):1138-48.
89. Neil ST, Kilbride M, Pitt L, Nothard S, Welford M, Sellwood W, Morrison AP. The questionnaire about the process of recovery (QPR): a measurement tool developed in collaboration with service users. Psychosis. 2009 Aug 1;1(2):145-55.
90. Kay SR, Fiszbein A, Opler LA. The positive and negative syndrome scale (PANSS) for schizophrenia. Schizophrenia bulletin. 1987 Jan 1;13(2):261-76.
91. Hermes ED, Sokoloff DM, Stroup TS, Rosenheck RA. Minimum clinically important difference in the Positive and Negative Syndrome Scale using data from the CATIE Schizophrenia Trial. The Journal of clinical psychiatry. 2012 Apr;73(4):526.
92. Kay SR, Opler LA, Lindenmayer JP. Reliability and validity of the positive and negative syndrome scale for schizophrenics. Psychiatry research. 1988 Jan 1;23(1):99-110.
93. Wechsler D. Wechsler Abbreviated Scale of Adult Intelligence (WASI).
94. Davies G, Rae CL, Garfinkel SN, Seth AK, Medford N, Critchley HD, Greenwood K. Impairment of perceptual metacognitive accuracy and reduced prefrontal grey matter

For peer review only - http://bmjopen.bmj.com/site/about/guidelines.xhtml

volume in first-episode psychosis. Cognitive neuropsychiatry. 2018 Feb 27:1-5.

95. Thoemmes F, MacKinnon DP, Reiser MR. Power analysis for complex mediational designs using Monte Carlo methods. Structural Equation Modeling. 2010 Jul 13;17(3):510-34.
96. McKnight PE, McKnight KM, Sidani S, Figueredo AJ. Missing data: A gentle introduction. Guilford Press; 2007 Mar 28.
97. Dong Y, Peng CY. Principled missing data methods for researchers. SpringerPlus. 2013 Dec 1;2(1):222.
98. Schafer JL, Graham JW. Missing data: our view of the state of the art. Psychological methods. 2002 Jun;7(2):147.
99. Allison PD. Missing data techniques for structural equation modeling. Journal of abnormal psychology. 2003 Nov;112(4):545.

AUTHOR'S CONTRIBUTIONS

Abigail Wright designed the study, supervised by Dr Kathryn Greenwood and Professor David Fowler.

FUNDING STATEMENT

This work was supported by Sussex Partnership NHS Foundation Trust and Economic Social Research Council, through a PhD studentship awarded to the first author (Reference: ES/J500173/1).

CONFLICTS OF INTERESTS STATEMENT

There is no conflict of interest.

ACKNOWLEDGEMENT

We thank Dr Samuel Berens who assisted in the design and coding of the Matlab tasks. We thank the Patient and Public Involvement forum, Psychosis Theme Group (PTG), who reviewed and commented on the project, in order to provide a lived experience perspective. The Sussex Psychosis Research Interest Group and Sackler Centre for Consciousness Science have also reviewed the original protocol.

For peer review only - http://bmjopen.bmj.com/site/about/guidelines.xhtml

Figure legends

Figure 1: Proposed model including associations between perceptual signal detection bias and metacognitive efficiency with various anomalous events: anomalous experiences and beliefs, and the associations between metacognitive aspects.

Figure 2: Part of figure 1 model. Proposed associations between metacognitive efficiency and metacognitive experience with monitoring and controlling processes.

Figure 3: Part of figure 1 model. Proposed associations between metacognitive experience and metacognitive knowledge with monitoring and controlling processes.

Figure 4: Proposed model for cross-sectional and longitudinal analyses including indirect effects from symptoms and neurocognition to metacognition and functional capacity to functional and subjective outcome.

For peer review only

For peer review only - http://bmjopen.bmj.com/site/about/guidelines.xhtml

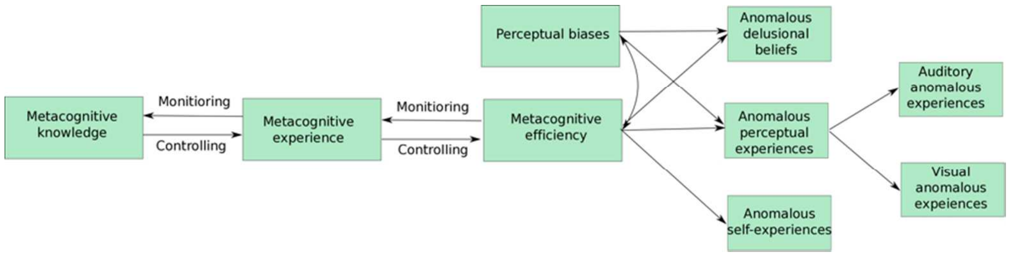

Figure 1: Proposed model including associations between perceptual signal detection bias and metacognitive efficiency with various anomalous events: anomalous experiences and beliefs, and the associations between metacognitive aspects.

159x39mm (150 x 150 DPI)

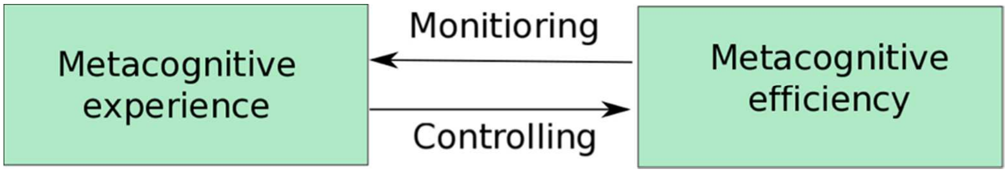

Figure 2: Part of figure 1 model. Proposed associations between metacognitive efficiency and metacognitive experience with monitoring and controlling processes.

159x26mm (150 x 150 DPI)

For peer review only

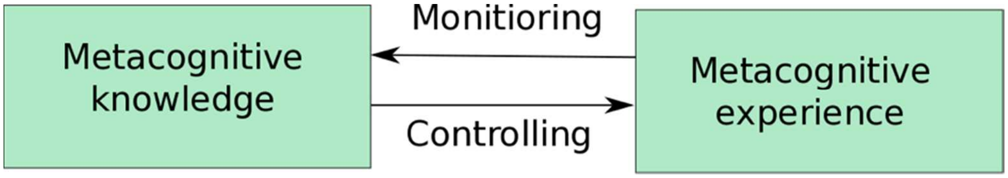

Figure 3: Part of figure 1 model. Proposed associations between metacognitive experience and metacognitive knowledge with monitoring and controlling processes.

159x27mm (150 x 150 DPI)

For peer review only

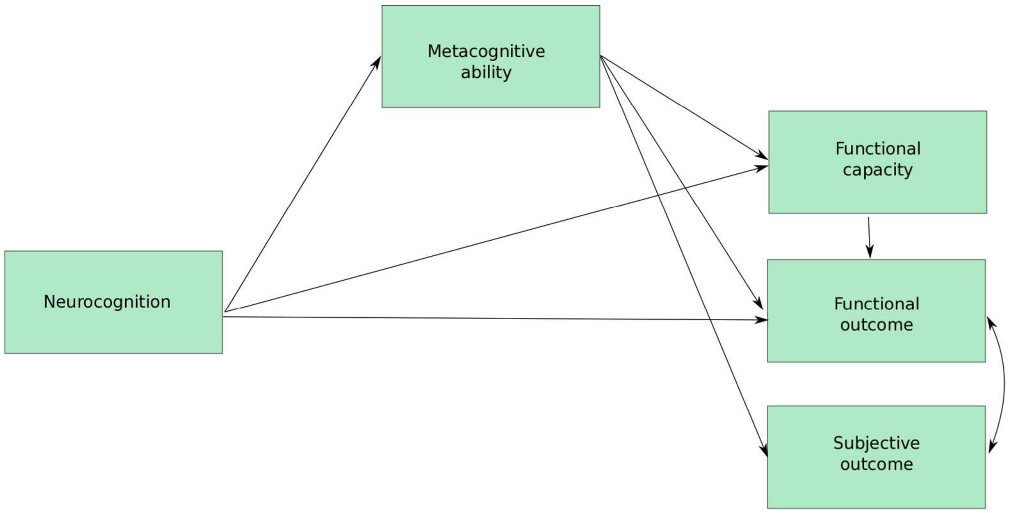

Figure 4: Proposed model for cross-sectional and longitudinal analyses including indirect effects from symptoms and neurocognition to metacognition and functional capacity to functional and subjective outcome.

632x318mm (96 x 96 DPI)

review only
